# A Novel Proline-Rich Cathelicidin from the Alpaca *Vicugna pacos* with Potency to Combat Antibiotic-Resistant Bacteria: Mechanism of Action and the Functional Role of the *C*-Terminal Region

**DOI:** 10.3390/membranes12050515

**Published:** 2022-05-12

**Authors:** Pavel V. Panteleev, Victoria N. Safronova, Roman N. Kruglikov, Ilia A. Bolosov, Ivan V. Bogdanov, Tatiana V. Ovchinnikova

**Affiliations:** M.M. Shemyakin & Yu.A. Ovchinnikov Institute of Bioorganic Chemistry, The Russian Academy of Sciences, Miklukho-Maklaya str., 16/10, 117997 Moscow, Russia; victoria.saf@ibch.ru (V.N.S.); kruglikov1911@mail.ru (R.N.K.); bolosov@ibch.ru (I.A.B.); contraton@mail.ru (I.V.B.); ovch@ibch.ru (T.V.O.)

**Keywords:** antimicrobial peptide, cathelicidin, proline-rich peptide, translation inhibitor, bacterial resistance, SbmA transporter

## Abstract

Over recent years, a growing number of bacterial species have become resistant to clinically relevant antibiotics. Proline-rich antimicrobial peptides (PrAMPs) having a potent antimicrobial activity and a negligible toxicity toward mammalian cells attract attention as new templates for the development of antibiotic drugs. Here, we mined genomes of all living *Camelidae* species and found a novel family of Bac7-like proline-rich cathelicidins which inhibited bacterial protein synthesis. The *N*-terminal region of a novel peptide from the alpaca *Vicugna pacos* named VicBac is responsible for inhibition of bacterial protein synthesis with an IC_50_ value of 0.5 µM in the *E. coli* cell-free system whereas the *C*-terminal region allows the peptide to penetrate bacterial membranes effectively. We also found that the full-length VicBac did not induce bacterial resistance after a two-week selection experiment, unlike the *N*-terminal truncated analog, which depended on the SbmA transport system. Both pro- and anti-inflammatory action of VicBac and its *N*-terminal truncated variant on various human cell types was found by multiplex immunoassay. The presence of the C-terminal tail in the natural VicBac does not provide for specific immune-modulatory effects in vitro but enhances the observed impact compared with the truncated analog. The pronounced antibacterial activity of VicBac, along with its moderate adverse effects on mammalian cells, make this molecule a promising scaffold for the development of peptide antibiotics.

## 1. Introduction

The discovery of antibiotics in the 20th century has revolutionized many areas of medicine, but the misuse of these compounds in recent decades has led to the spread of resistant strains of bacteria [1,2,3]. About half of the known antibiotics affect the translation machinery of target cells and therefore are of great importance for medicine. Novel ribosome inhibitors should be highly effective against a wide range of pathogens and also should have a mechanism of action different from those of known antibiotics to prevent the cross-resistance effects. One of the classes of the desired compounds, which in recent years attracted much attention from scientists, are proline-rich antimicrobial peptides (PrAMPs) [4,5]. Despite moderate amino acid sequence homology and different polypeptide chain length, PrAMPs bind to an overlapping site within the ribosomal exit tunnel and inhibit translation either by blocking the transition from the initiation to the elongation phase [6,7] or by preventing dissociation of the termination factors [8]. Notably, PrAMPs have multiple contacts in the ribosomal exit tunnel [6,9], which minimizes the risk of bacterial resistance development due to modifications in rRNA and prevents cross-resistance in strains tolerant to small-molecule antibiotics with similar binding sites, such as macrolides. Low probability of cross-resistance, the presence of effects of collateral sensitivity and synergy with human host-defense peptides [10], as well as relatively low cytotoxicity compared to most other membranotropic antimicrobial peptides and the lack of immunogenicity [11] allow considering PrAMPs as promising molecular scaffolds for the development of new antibiotics. Due to high proline content, these AMPs are likely to be more resistant to serine proteases, elastase, and aminopeptidases, which results in a longer half-life [12]. In addition, compared to traditional antibiotics, the use of PrAMPs provides a long-term post-antibiotic effect when used at insignificantly higher concentrations than values of the minimum inhibitory concentration (MIC) [13].

The known insect PrAMPs are low-hydrophobic molecules and, therefore, they are practically unable to penetrate bacterial membranes of target cells without specific carrier proteins or co-expressed membrane-active AMPs of the host organism [14,15,16]. Recent studies identified two key transporters responsible for PrAMP uptake—SbmA [17] and MdtM [18]. Mammalian proline-rich cathelicidins usually have a length of more than 30 amino acid residues with a hydrophobic *C*-terminal region and, therefore, can effectively cross the outer and the cytoplasmic membranes of bacteria and even damage them at higher concentrations switching to the membranolytic mechanism of action. Artiodactyla species are large natural reservoirs of cathelicidins relative to other mammals and, in particular, to humans expressing only one cathelicidin, known as LL-37. Earlier, a set of cathelicidins, including PrAMPs, were mined in genomes of different Artiodactyla and Cetacea species [7,19,20,21]. In this work, we used genome mining to identify novel families of PrAMPs in living species of the *Camelidae* family. The first representatives of this family appeared early in the evolution of Artiodactyls, around 60 to 40 million years ago during the middle Eocene period [22]. In addition, *Camelidae* species have unique immunological features that are not observed in other mammalians [23]. Therefore, a unique panel of cathelicidin structure scaffolds was expected as well. As a result, several different structural subgroups were identified, some of them belonging to the α-helical lysine/arginine-rich myeloid family, prophenin-like family, and a few other novel proline-rich families. Next, we performed a structure-activity relationship study of the novel 39-residue PrAMP from the alpaca *Vicugna pacos* designated as VicBac. The high similarity of VicBac to Bac7 in its *N*-terminal part suggested a ribosome-targeting mechanism of action. At the same time, this peptide has no regular structure patterns in the *C*-terminal part, which is in sharp contrast to other known PrAMPs from artiodactyls. A comprehensive comparative study of the wild-type VicBac and its shortened derivatives was carried out: the peptides were obtained and characterized regarding their antimicrobial activity, mode of action against bacteria, cytotoxicity, and immunomodulatory properties.

## 2. Materials and Methods

### 2.1. Identification of CATHL Genes in Camelidae WGS Database

The TBLASTN program was used to identify cathelicidin genes in the whole-genome shotgun (WGS, GenBank) database using conservative cathelin-like domain (CLD) fragment FTVKETVCPRTSPQPPEQCDFKE encoded by nucleotide sequence located in the second exon of the cattle procathelicidin-3 (Bac7 precursor, GenBank: NP_776426.1) as a query against all Camelidae WGS projects deposed in NCBI using the values of the default parameters (matrix: BLOSUM62, gap costs: existence 11, extension 1). Then, the obtained hit DNA contigs (±2000 bp relative to the query) were analyzed by the GenScan program (http://genes.mit.edu/GENSCAN.html; last accessed 10 March 2022) to identify exons within the genomic sequence. The putative elastase processing sites in the fourth exon were suggested based on information about known Cetartiodactyla cathelicidins [19]. Finally, putative mature cathelicidin sequences were manually (visually) inspected and additionally analyzed by CAMP database instruments (http://www.camp.bicnirrh.res.in/index.php; last accessed 10 March 2022) to identify known cathelicidins having the highest amino acid sequence similarity with found ones.

### 2.2. Expression and Purification of the Antimicrobial Peptides

The recombinant plasmids for the expression of proline-rich cathelicidins were constructed using a pET-based vector as described previously [20]. The coding sequences were designed based on *E. coli* codon usage bias data. The expression cassette was composed of the T7 promoter, the ribosome binding site, and the sequence encoding the chimeric protein that included octahistidine tag, the *E. coli* thioredoxin A with the M37L substitution (TrxL), methionine residue, and cathelicidin. *E. coli* BL21 (DE3) cells were transformed with the corresponding plasmids and grown up from an initial OD_600_ of 0.01 for 24 h at 30 °C with a speed of 220 rpm in ZYP-5052 auto-inducing medium based on lysogeny broth (LB) supplemented with 0.2% lactose, 0.05% glucose, 0.5% glycerol, 1 mM MgSO_4_, 50 mM Na_2_HPO_4_, 50 mM KH_2_PO_4_, 25 mM (NH_4_)_2_SO_4_, 100 μg/mL of ampicillin, and trace metals according to Studier [24]. The cultured cells were harvested by centrifugation and sonicated in the 100 mM phosphate buffer (pH 7.8) containing 20 mM imidazole and 6 M guanidine hydrochloride. The clarified lysate was loaded on a column packed with Ni Sepharose (GE Healthcare, Chicago, IL, USA). The recombinant protein was eluted with the buffer containing 0.5 M imidazole. The eluate was acidified (up to pH 1.0) by the concentrated hydrochloric acid, and the fusion protein was cleaved by a 100-fold molar excess of CNBr over methionine at 25 °C for 18 h in the dark. The lyophilized products of the cleavage reaction were dissolved in water and loaded on a semi-preparative Reprosil-pur C_18_-AQ column (10 × 250 mm^2^, 5-μm particle size, Dr. Maisch GmbH; Ammerbuch-Entringen, Germany). Reversed-phase high-performance liquid chromatography (RP-HPLC) was performed with a linear gradient of acetonitrile in water containing 0.1% TFA. The peaks were monitored at 214 and 280 nm (Figure S1), collected and analyzed by MALDI-TOF MS using Reflex III mass-spectrometer (Bruker Daltonics, Bremen, Germany). The obtained fractions with corresponding molecular masses (Table S1) were dried in vacuo and dissolved in water. Human cathelicidin LL-37, *C*-terminally amidated 39-residue full-length VicBac, and melittin (>98% pure for all the peptides) synthesized using a standard solid-phase method were kindly provided by Dr. Maxim N. Zhmak and Dr. Sergey V. Sychev (M.M. Shemyakin and Yu.A. Ovchinnikov Institute of Bioorganic Chemistry of the Russian Academy of Sciences, Moscow, Russia).

### 2.3. Bacterial Strains

The following reference strains were utilized: *Escherichia coli* BL21 (DE3), *E. coli* DH10B, *E. coli* ML-35p, *E. coli* ATCC 25922, *Pseudomonas aeruginosa* ATCC 27853, *P. aeruginosa* PAO1, *Staphylococcus aureus* ATCC 6538P, *S. aureus* ATCC 29213, *Bacillus subtilis* B-886, *B. licheniformis* VKM B511, *Mycobacterium phlei* Ac-1291. Strain *E. coli* BW25113 and its knockout variants Δ*sbmA*, Δ*mdtM*, Δ*ompF* from the KEIO collection [25] were kindly provided by Dr. Ilya A. Osterman. The clinical isolates were collected and provided by Sechenov First Moscow State Medical University hospital and Solixant LLC (Moscow, Russia). The detailed characteristics of utilized clinical isolates are presented in [20].

### 2.4. Antimicrobial Assay

Bacterial test cultures were grown in the Mueller-Hinton broth (MH; Sigma, St. Louis, MO, USA) at 37 °C to mid-log phase and then diluted with the 2× MH medium supplemented with 1.8% NaCl or without salt so that to reach a final cell concentration of 10^6^ CFU/mL. 50 µL of the obtained bacterial suspension were added to aliquots of 50 µL of the peptide solutions serially diluted with sterilized 0.1% bovine serum albumin (BSA) in 96-well flat-bottom polystyrene microplates (Eppendorf #0030730011, Hamburg, Germany). After incubation for 24 h at 37 °C and 950 rpm on the plate thermoshaker (Biosan, Riga, Latvia), minimum inhibitory concentrations (MIC) were determined as the lowest peptide concentrations that prevented the growth of a test microorganism observed as visible turbidity, and the absorbance at 620 nm. To verify MIC values, the respiratory activity of the bacteria was determined. Briefly, 20 µL of 0.1 mg/mL resazurin (a redox indicator; Sigma, St. Louis, MO, USA) was added to the wells after 24 h of incubation, and the plate was incubated for an additional two hours. The reduction of resazurin to resorufin was measured. In most cases, no significant divergence of MIC values was observed (within ±1 dilution step). The results were expressed as the median values determined based on at least three independent experiments performed in triplicate. 

### 2.5. In Vitro Transcription/Translation Inhibition Assay

To investigate the effect of cathelicidins on the coupled transcription/translation process, the peptides were added to a cell-free protein synthesis (CFPS) reaction mix with a plasmid encoding enhanced green fluorescent protein (EGFP) variant (F64L, S65T, Q80R, F99S, M153T, and V163A) under control of the T7 promoter as described previously with some modifications [20]. Briefly, the peptides were dissolved in 0.1% BSA in water. Bac7[1–22] was used as a positive control inhibitor. The fluorescence of the sample without inhibitor was set as the 100% value. The reaction proceeded for 2 h in 96-well v-bottom black polystyrene microplates in a plate shaker (30 °C, 1000 rpm). The fluorescence of the synthesized EGFP was measured with the microplate reader AF2200 (λ_Exc_ = 488 nm, λ_Em_ = 510 nm; Eppendorf, Hamburg, Germany). The experimental data were obtained from at least two independent experiments performed in triplicate. Non-linear regression curves were generated using GraphPad Prism v.8.0.1 (GraphPad Software Inc., San Diego, CA, USA). 

### 2.6. Assessment of Bacterial Membrane Permeabilization

To examine the ability of the peptides to affect the barrier function of the cytoplasmic bacterial membrane, we slightly modified the previously described procedure [20] with the use of the *E. coli* ML-35p strain constitutively expressing cytoplasmic β-galactosidase but lacking lactose permease. The state of the *E. coli* ML-35p inner membrane was assessed based on its permeability to chromogenic marker *o*-nitrophenyl-β-D-galactopyranoside (ONPG, AppliChem, Darmstadt, Germany), which is the β-galactosidase substrate. The cells were incubated in the trypticase soy broth (TSB) for 16 h at 37 °C, washed three times with phosphate-buffered saline (PBS, pH 7.4) to remove residual growth media, adjusted to the concentration of 2.5 × 10^8^ CFU/mL, and stored on ice until used. The assay was performed in PBS as well. The final concentration of *E. coli* ML-35p cells was 2.5 × 10^7^ CFU/mL. The concentration of ONPG was 2.5 mM. Peptide samples were placed in the wells of a 96-well plate with a non-binding surface (NBS, Corning #3641), and the optical density (OD) of the solution rose due to the appearance of o-nitrophenol was measured at 405 nm using a microplate reader AF2200 (Eppendorf, Hamburg, Germany). The final volume in each well was 200 µL. Assays were performed at 37 °C under stirring at 500 rpm. Control experiments were performed under the same conditions without adding a peptide. Two independent experiments were performed, and the curve pattern was similar. 

### 2.7. Resistance Induction Experiments

Resistance induction experiments were performed using the previously described method [20]. Briefly, on day one, the overnight culture of wild-type bacteria was diluted with the 2× MH broth supplemented with 1.8% NaCl to reach a final cell concentration of 10^6^ CFU/mL. 50 µL of the obtained bacterial suspension were added to aliquots of 50 µL of the peptide solutions serially diluted with the sterilized 0.1% BSA in 96-well flat-bottom polystyrene microplates. After incubation for 20 ± 2 h at 37 °C and 950 rpm, MICs were determined as described above. For each subsequent daily transfer, 2–4 μL of the inoculum taken from the first well containing a sub-inhibitory drug concentration were diluted with 2 mL of the fresh 2× MH broth supplemented with 1.8% NaCl. Then, 50 µL of this suspension were sub-cultured into the next passage wells containing 50 µL aliquots of the peptide at concentrations from 0.25× to 8–16× of the current MIC of each agent. 14 repeated passages in the presence of antimicrobial agents were made for each bacterial strain during the experiment. Bacteria that grew at the highest concentration of AMPs on the final day were passaged a further 3 times on drug-free agar plates before determining the final MIC value. Control serial passages in the absence of the agent were also included, and the resulting cultures showed unchanged MICs against antibacterial agents. 

### 2.8. Whole-Genome Sequencing

To identify potential mechanisms conferring resistance to VicBac[1–22], we performed whole-genome sequencing of resistant strain followed by genomic DNA *de novo* assembly and variant calling. Assembled wild-type *E. coli* MDR 1057 strain genome was used as reference. 2 × 150 bp pair-end sequencing of prepared genomic DNA was performed with an Illumina MiSeq platform (Illumina, San Diego, CA, USA). Evaluation of read quality was performed using FastQC software (v0.11.9) [26], then reads were filtered, and adapters were cut with TrimmomaticPE (v0.39) [27]. SPAdes software (v3.13.0) was used to assemble genomes utilizing both filtered paired-end and unpaired reads [28]. Assembly quality was then evaluated with the QUAST program (v5.0.2) [29]. Gene prediction and annotation of assembled contigs were made with the Prokka program (v1.14.6) [30]. Alignment of paired-end reads on reference genome was made using BWA-MEM (v0.7.17-r1188) algorithm [31]. To call actual variants, VarScan software (v2.4.0) was launched with a minimal reported variant frequency set to 0.9 [32].

### 2.9. Molecular Cloning Procedures

To prove the obtained whole-genome sequencing data, the *sbmA* gene, as well as its regulatory part, was amplified by polymerase chain reaction (PCR) using specific primers as described previously [20]. Briefly, individual bacterial colonies of the tested strain were picked up and used as a template for PCR. The PCR products were inserted into the pAL-2T vector (Evrogen, Moscow, Russia). The ligation products were transformed into the chemically competent *E. coli* DH10B cells. The obtained plasmids were sequenced on both strands using the ABI PRISM 3100-Avant automatic sequencer (Applied Biosystems, Foster City, CA, USA). At least two independent experiments were performed.

A plasmid vector for the expression of sfGFP under the strong constitutive artificial promoter J23119 [33] was kindly provided by M.N. Baranova (Figure S2A). This plasmid was used for the preparation of the complementation plasmids overexpressing two SbmA variants in the SbmA-deficient *E. coli* strain. The plasmids were obtained by ligase-independent cloning procedure [34]. Briefly, DNA parts were produced by PCR-amplification of the vector and target *sbmA* gene from wild-type *E. coli* MDR 1057 or VicBac[1–22]-resistant strain (Figure S2B). The DNA fragments purified by gel electrophoresis and having 22–23 bp overhangs were mixed with molar ratios of 2:1 (insert: linearized vector) and were subsequently transformed into chemically competent *E. coli* DH10B cells. Target plasmids were isolated from individual clones and then analyzed by DNA sequencing.

### 2.10. Hemolysis and Cytotoxicity Assay

Hemolytic activity of the peptides was tested against the fresh suspension of human red blood cells (hRBC) using the hemoglobin release assay as described previously [35]. Two experiments were performed with the hRBC from blood samples of independent donors. The quantitative data were represented as average means with standard deviations. The colorimetric 3-(4,5-dimethylthiazol-2-yl)-2,5-diphenyltetrazolium bromide (MTT) dye reduction assay was used to determine the cytotoxicity of the peptides against transformed human embryonic kidney cells (HEK293T). 10^4^ cells per well in Dulbecco’s modified Eagle’s medium (DMEM/F12) supplemented with 10% fetal bovine serum (FBS; Invitrogen, Waltham, MA, USA) were placed into 96-well plates and then cultured in the CO_2_-incubator (5% CO_2_, 37 °C). After the media were removed, the peptides were dissolved in 100 µL of the same medium and added to cell cultures at different final concentrations. 20 h later, 20 μL of MTT (5 mg/mL; Sigma, St. Louis, MO, USA) was added to each well, and the plates were incubated for 4 h at 37 °C. Then, the media were discarded and a 100 µL dimethyl sulfoxide-isopropanol mixture at a ratio of 1:1 (*v*/*v*) was added to each well to dissolve the crystallized formazan. The absorbance at 570 nm was measured by a microplate reader AF2200 (Eppendorf, Hamburg, Germany). The optical density in the wells containing cells cultured without the peptides was assumed to represent 100% cell viability. Two independent experiments were performed for each peptide.

### 2.11. Cytokine Response to Cathelicidins on Human Cells In Vitro

Acute monocytic leukemia THP-1 line (ATCC TIB-202) was cultured in complete RPMI 1640 medium (Invitrogen, Waltham, MA, USA) containing 10% FBS, 1× antibiotic-antimycotic solution (Invitrogen, Waltham, MA, USA), and 0.05 mM β-mercaptoethanol, in the CO_2_-incubator (5% CO_2_, 37 °C). THP-1 cells were differentiated into proinflammatory macrophages (MΦ1) according to the previously reported protocol [36]. Primary peripheral blood mononuclear cells (PBMC) collected from a healthy donor were purchased from American Type Culture Collection (ATCC PCS-800-011), thawed, and seeded into a 96-well plate one day prior to the experiment at a density of 2 × 10^5^ cells/well. Two different cell subpopulations (monocytes and T-/B-/NK-lymphocytes) were isolated from PBMC based on their adherence ability. Macrophages MΦ1 were washed out and seeded into 96-well plates at a density of 10^5^ cells/well one day prior to the experiment. The next day medium in each well was replaced by a fresh complete RPMI 1640 medium with or without 2 μM of cathelicidin (VicBac[1–22] or VicBac[1–39]). Cell cultures were kept in a CO_2_ incubator (5% CO_2_, 37 °C) for 24 h. Culture supernatants were collected 24 h later and stored at −70 °C degrees less than one week prior to the assessment of analytes. 27 analytes were measured at a protein level by multiplex xMAP technology using the MILLIPLEX MAP Human Cytokine/Chemokine Magnetic Bead Immunology Panel kit (HCYTOMAG-60K-27, Merck, Darmstadt, Germany): eotaxin-1/CCL11, TGFα, GM-CSF, IFNα2, IFNγ, IL-10, IL-12p40, IL-12p70, IL-13, IL-15, sCD40L, IL-17A, IL-1RA, IL-1α, IL-9, IL-1β, IL-2, IL-3, IL-4, IL-5, IL-6, IL-7, IL-8/CXCL8, IP-10/CXCL10, RANTES/CCL5, TNFα, TNFβ. Multiplex-based assay read-out was performed using the MAGPIX system (Merck) with the xPONENT 4.2 software (Merck, Darmstadt, Germany) in accordance with the manufacturer’s instruction with overnight incubation of the samples with primary antibodies. The final analysis was carried out with the MILLIPLEX Analyst v5.1 software (Merck). Measurements were performed twice for each sample. The release of the analytes in control and experimental samples were compared by unpaired two-sample *t*-test using GraphPad Prism v.8.0.1. The *p* values ≤ 0.05 were considered significant.

## 3. Results and Discussion

### 3.1. Identification of Novel Proline-Rich Cathelicidins in Camelidae Species

**Figure 1:**
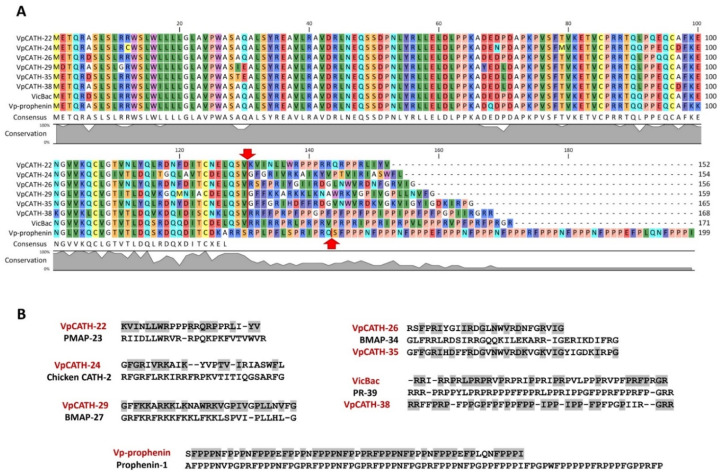
(**A**) Amino acid sequence alignment of preprocathelicidins from the alpaca *Vicugna pacos*. The alignment was made using the CLC Sequence Viewer software (version 8.0). The putative post-translational processing sites are marked with a red arrow. (**B**) Amino acid sequence alignment of mature cathelicidins from the alpaca with known cathelicidins from the pig *Sus scrofa* (PMAP-23, PR-39, prophenin-1), chicken *Gallus domesticus* (CATH-2), and cow *Bos taurus* (BMAP-27, BMAP-34).

In contrast to invertebrate species, PrAMPs are likely to be common host-defense peptide weapons in artiodactyl mammals. These peptides are synthesized as a part of gene clusters encoding panels of cathelicidins [37]. The precursors of cathelicidins are characterized by the presence of the cathelin-like domain (CLD, 99-114 amino acid residues)—the conserved pro-region encoded by the first three exons. Highly variable mature cathelicidins are located at the *C*-terminus and encoded by the fourth exon. This allows to carry out a bioinformatic search using sequences of conservative fragments of the cathelin-like domain and WGS databases. In this study, we aimed to identify novel families of proline-rich ribosome inhibitors in *Camelidae* mammalians that have not been screened yet. With this in view, seven currently living species (the Arabian camel *Camelus dromedarius*, the Mongolian camel *C. bactrianus*, the wild Bactrian camel *C. ferus*, the llama *Lama glama*, the guanaco *Lama guanicoe*, the alpaca *Vicugna pacos*, and the vicuna *V. vicugna*) were analyzed. As a result, 62 cathelicidins, including 35 unique peptides, were identified using the genome mining approach (Table S2). These cathelicidins were classified into 9 different structural subgroups, with some of them belonging to the lysine/arginine-rich myeloid family (α-helical MAP-like peptides, Figure S3) and having mature peptide lengths from 23 to 35 amino acid residues, and also pertaining to the prophenin-like family with the length variation from 26 to 116 residues. Bioinformatic analysis revealed that some novel families did not share significant similarity with any known peptides listed in different AMP databases, in particular: (1) short 22-residue proline-rich peptides having a partial similarity with the porcine PMAP-23 in the *N*-terminal region, (2) 38-residue proline-rich peptides with unique (PPX)_4_ motif in its central part, and (3) 41-residue proline-rich peptides similar to the bovine Bac7 or to the porcine PR-39 in its *N*-terminal region. A detailed analysis of the gene organization (Figure S4), preprocathelicidin structure (Figure 1A), as well as sequence alignment with known AMPs (Figure 1B) were performed for the panel of cathelicidins from the alpaca *V. pacos*. The genes encoding found peptides displayed all the characteristics of a functional cathelicidin, including (1) a gene size of about 2 kb, (2) a conserved four exons/three introns arrangement with intact splicing sites, (3) TATA-box immediately upstream from the transcription start site, (4) a polyadenylation signal located about 60–120 bp away from the stop codon (Figure S4).

We found 8 sequences of 9 novel cathelicidin subgroups in the alpaca genome, which were designated as VpCATH-22 (proline-rich), VpCATH-24 (α-helical MAP-like), VpCATH-26 (α-helical MAP-like), VpCATH-29 (α-helical MAP-like), VpCATH-35 (α-helical MAP-like), VpCATH-38 (proline-rich), Vp-prophenin (proline-rich), and VicBac (proline-rich).

**Figure 2:**
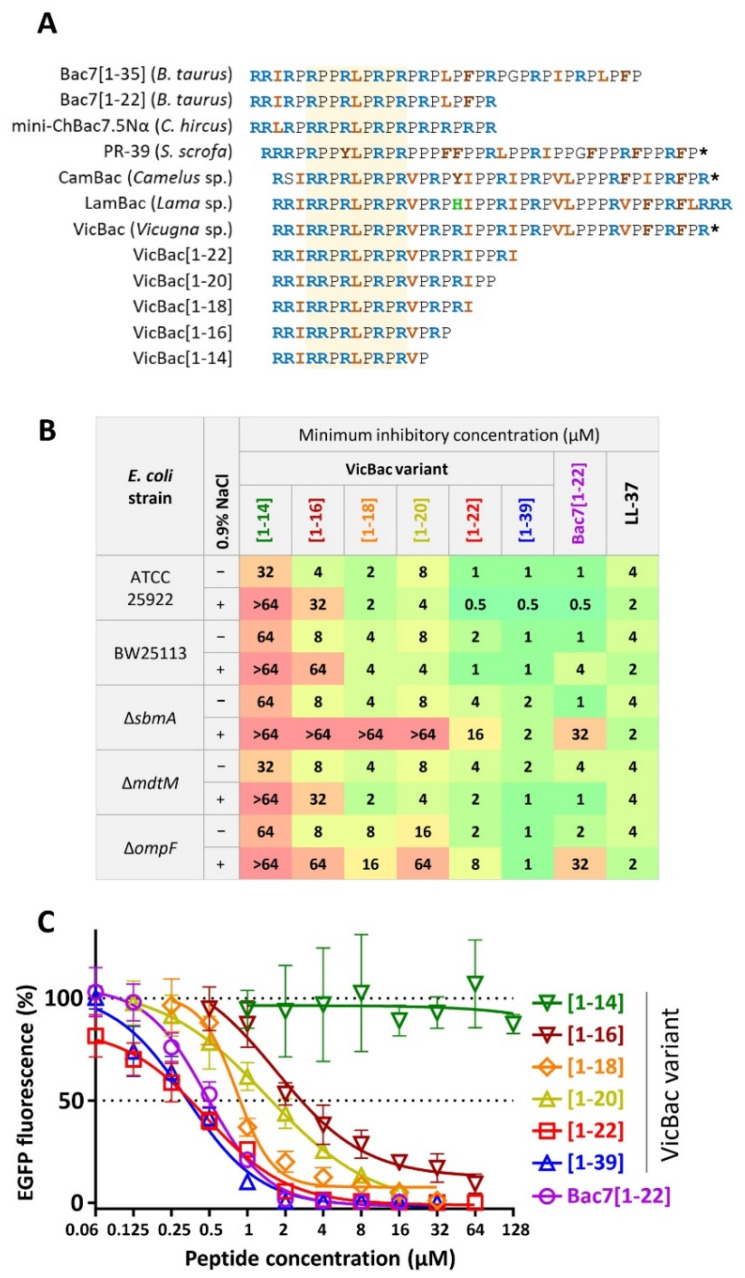
(**A**) Amino acid sequence alignment of VicBac, its orthologs from camels (CamBac) and llamas (LamBac), and o``ther known proline-rich cathelicidins. A consensus sequence that is essential for the inhibition of the protein synthesis by oncocin- and Bac7-like PrAMPs is marked with yellow shading. A *C*-terminal amidation is marked with an asterisk (*). (**B**) Antibacterial activity of the wild-type amidated VicBac[1–39], its truncated variants, and known cathelicidins in the rich Mueller–Hinton broth ±0.9% NaCl. (**C**) Effects of VicBac[1–39], its truncated variants, and Bac7[1–22] at different concentrations on the fluorescence resulting from the *in vitro* coupled transcription/translation of EGFP with the use of the *E. coli* BL21 (DE3) Star cell extract. Data are the mean ± SD of at least two independent experiments performed in triplicate.

The *N*-terminal fragment [1–16] of Bac7 retains an ability to inhibit translation with a high efficiency comparable to that of the full-length peptide [6]. Notably, VicBac contains a consensus fragment (R/K)XX(R/Y)LPRPR required for protein biosynthesis inhibition by oncocin- and Bac7-like PrAMPs and their strong binding in the nascent peptide exit tunnel of 70S ribosome (Figure 2A) [4,38]. Together, a high similarity of VicBac to Bac7 in their *N*-terminal parts suggested a ribosome-targeting mechanism of action. The amino acid sequence alignment of VicBac and the known PrAMPs also allows one to suggest a shorter protrusion length of the new peptide in the ribosome A-site binding pocket (Figure 2A). Notably, three orthologs of this subgroup were found: VicBac, CamBac, LamBac from *Vicugna* sp., *Camelus* sp., and *Lama* sp., respectively (Figure 2A). The latter one has no additional post-translational modifications, while others bear the *C*-terminal GR dipeptide that seems to be cleaved by the enzyme peptidylglycine alpha-amidating monooxygenase (PAM) followed by amidation of the carboxyl group of the preceding amino acid residue. Interestingly, this new proline-rich subgroup has no regular structure patterns in the *C*-terminal part, which is in a sharp contrast to Bac7 and PR-39 (or Bac5) having (PX)_n_ and (XXPP)_n_ motifs, respectively.

### 3.2. N-Terminal Fragments of Proline-Rich Cathelicidin VicBac Inhibit Protein Biosynthesis in Bacteria

To find minimal fragments that retain antimicrobial activity and inhibit bacterial translation, a set of peptides were synthesized by shortening VicBac from the *C*-terminus (Figure 2A). The *C*-terminal residues seem to be less significant for the antimicrobial activity of PrAMPs, as shown for Bac7 and Tur1A [7,39]. In contrast, the deletion of several residues from the *N*-terminus of oncocins and Bac7, which are located in the A-site binding pocket, strongly reduces antimicrobial activity and underlines the crucial role of this structural element [6,15,39]. To obtain VicBac truncated analogs, we selected a heterologous expression system using thioredoxin A as a carrier protein. Thioredoxin has a size close to that of the cathelin-like domain and was approved to be an effective carrier protein for PrAMPs in our previous studies [20]. The peptides were purified by a downstream process, including IMAC of the clarified total cell lysate, cleavage of the fusion protein with cyanogen bromide, and fine purification by RP-HPLC (Figure S1). The final yields of the peptides were from 4 to 10 mg per 1 L of the culture medium. To obtain full-length amidated VicBac (also referred to as [1–39]), the solid-phase synthesis technique was utilized.

The microdilution assay revealed that the analog [1–22] fully retained activity against the *E. coli* strains ATCC25922 and BW25113, thus indicating that the *N*-terminal region is indeed crucial for the VicBac antimicrobial activity (Figure 2B). These findings are consistent with the data on inhibition of in vitro protein synthesis in the *E. coli* cell-free system—both peptides, as well as the Bac7 analog used as control, demonstrated similar inhibition curves with an IC_50_ value of ~0.5 µM (Figure 2C). Notably, almost complete inhibition of translation by the peptides is achieved at concentrations equal to the MIC values against *E. coli*. Thus, the *C*-terminus is not important for translational inhibition, which is similar to data obtained for Bac7. Further truncation of the peptide leads to a gradual decrease in activity, and the activity disappears when the length of the peptide is 14 residues. Therefore, the fragment [1–16] is minimal, providing both translation inhibition (IC_50_ value ~4 µM) and killing of *E. coli* at 4–8 µM under salt-free conditions. Surprisingly, the ability of the fragment [1–20] to inhibit translation and suppress cell growth is lower than that of [1–18]. It can be assumed that the absence of contacts between the terminal Pro-Pro motif and the ribosome, on the contrary, may interfere with the peptide binding as compared to the peptide [1–18]. The presence of the 0.9% NaCl strongly influences the activity of VicBac shortened analogs. Analogically, the shortened analog [1–16] of caprine PrAMP mini-ChBac7.5Na was shown to be more salt-sensitive as compared with the wild-type peptide [20].

Next, we analyzed the activity of the peptides against strains deficient in known PrAMP transporters (SbmA or MdtM) or in a key porin OmpF that provides transport for a number of peptide translation inhibitors [40]. It is interesting to note that in the absence of salt, there is no effect of these proteins on the activity of the peptides. On the contrary, under the physiological conditions, with the exception of the full-length VicBac, all the shortened peptides lost their activity against Δ*sbmA* and Δ*ompF* strains. This suggests that in such an environment, all the fragments endowed with antimicrobial potential exploit the same internalization route via SbmA and OmpF, but not MdtM. Except for a slight 2-fold increase in MIC against Δ*sbmA* strain, the VicBac was demonstrated to be independent of known transporter proteins, which is also characteristic of the membranotropic cathelicidin LL-37 according to our data (Figure 2B). Here, we found that an antibacterial activity of the VicBac fragments with a length from 14 to 22 residues, in general, correlated with the data on translation inhibition. Therefore, the lack of activity seems to be a consequence of the lower capacity to inhibit the translational machinery rather than decreased cell uptake based upon transport via SbmA. Taking into account the high activity of the analog [1–22], along with that of the full-length VicBac, the former one can be considered as a basis for designing antibiotics.

### 3.3. The Biological Activity of Cathelicidin VicBac and Its Truncated Analog

**Figure 3:**
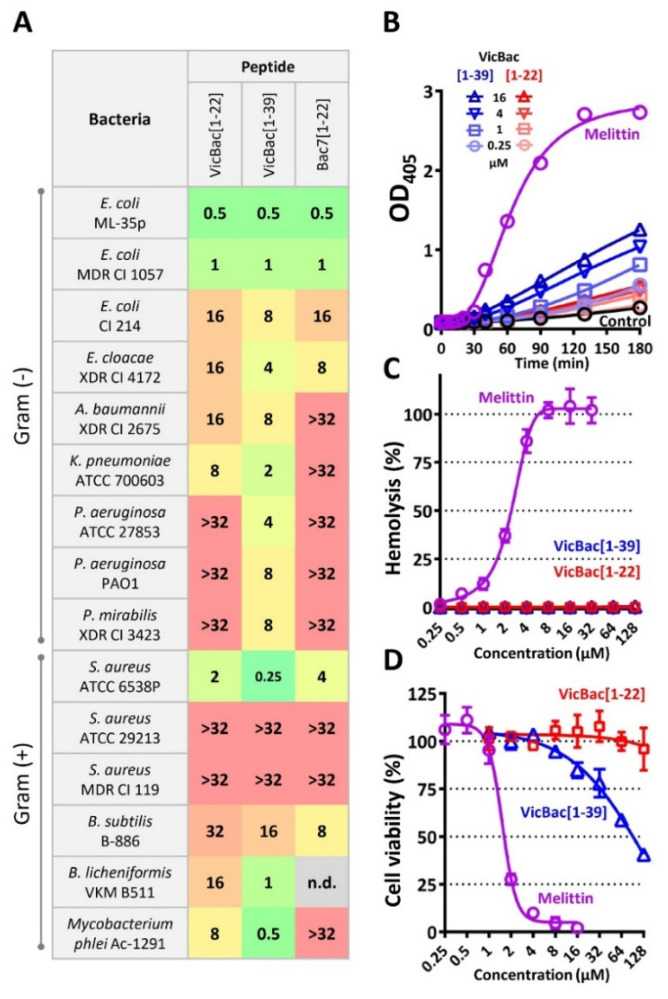
Therapeutic potential of the alpaca cathelicidin VicBac[1–39] and its *N*-terminal fragment [1–22]. (**A**) Antibacterial activity of the peptides in the rich Mueller-Hinton broth supplemented with 0.9% NaCl. (**B**) Kinetics of changes in *E. coli* ML-35p cytoplasmic membrane permeability measured using ONPG (OD_405_) hydrolysis. (**C**) Hemolytic activity after 1.5 h incubation (hemoglobin release assay). (**D**) Cytotoxicity against human embryonic kidney cells (HEK293T) after 20 h incubation (MTT-assay). The data are presented as the mean ± SD of two independent experiments.

Essential characteristics of any therapeutic drug are its target specificity and non-toxicity in vitro, providing its high therapeutic index or wide therapeutic window. The antibacterial activity of VicBac and its *N*-terminal fragment were determined using a two-fold serial dilution assay. The high ionic strength of the test medium is known to lower the antibacterial activity of PrAMPs, and here we used the Mueller–Hinton broth supplemented with 0.9% NaCl to analyze it. Minimum inhibitory concentrations (MICs) of the investigated peptides and the reference peptide Bac7[1–22] against Gram-positive and Gram-negative bacteria are presented in Figure 3A. The full-length VicBac displayed a pronounced inhibitory activity against a panel of reference bacterial pathogens as well as against antibiotic-resistant clinical isolates with MIC values which in most cases were in the low micromolar range from 0.25 to 8 µM. Similar to the dolphin Tur1A and the bovine Bac7[1–35] [21], the cathelicidin VicBac could not inhibit the *S. aureus* ATCC 25923. The shortened variant VicBac[1–22], as well as Bac7[1–22], had a narrower spectrum of antibacterial activity with the most pronounced effect against *E. coli* strains.

Previously described PrAMPs caused appreciable damage to bacterial membranes only at higher concentrations than their MICs. Spectrophotometric monitoring of the *E. coli* ML-35p cytoplasmic membrane permeability for the chromogenic marker ONPG revealed that the full-length VicBac demonstrated a quite moderate effect and acted in a dose-dependent manner while the shortened analog did not retain such an ability (Figure 3B). Similar results were obtained earlier for other mammalian PrAMPs like Tur1A and Bac7[1–35] [21]. Notably, VicBac[1–39] shows a negligible membrane damage effect (Figure 3B) at concentrations near the MIC value against this strain (0.5 µM, Figure 3A). Therefore, it is fair to assume that the *C*-terminal part facilitates penetration of the peptide inside the cell, bypassing specific transporters rather than causing membrane damage to *E. coli* and changing the mode of action. On the other hand, the elevated rate of membrane damage at the peptide concentrations of 8–16 µM after 3 h incubation may contribute to the activity of VicBac[1–39] against strains lacking SbmA transporters like *P. aeruginosa* or some Gram-positive bacteria. Both scenarios could be possible and depend on peptide concentration and membrane composition of bacteria. Expectedly, the control peptide melittin trigger to inflict significant damage to the inner membrane within the first 30 min.

To evaluate the cytotoxic effect of the peptides, human red blood cells (hRBC), as well as adhesive cell lines of human embryonic kidney cells (HEK293T), were used. Melittin, known as a powerful cytolytic agent, was used as a positive control. Both investigated peptides lacked hemolytic activity at concentrations up to 128 µM (Figure 3C), while melittin almost completely lysed all the cells tested at concentrations of <4 μM (Figure 3C,D). It is known that relatively short PrAMPs have no pronounced toxicity toward mammalian cells. Indeed, the data analysis revealed that VicBac, but not its shortened analog [1–22], showed moderate cytotoxic activity against human HEK293T cells with an IC_50_ value of ~100 μM (Figure 3D). Nevertheless, the observed cytotoxic effects become evident at concentrations >16 µM that, in any case, provide a significant therapeutic window against some highly susceptible bacteria like *E. coli* or *K. pneumoniae*.

### 3.4. The Presence of the C-Terminal Hydrophobic Motif Prevents Bacterial Resistance to VicBac

Last decade, it was becoming apparent that bacteria can evolve resistance to AMPs, although specific mechanisms of bacteria-killing by cationic AMPs are much more favorable than those of conventional antibiotics to prevent resistance evolution [41]. In particular, PrAMPs can interact with several conservative targets within bacterial cells like ribosomes [4] or DnaK [42], and therefore the probability of the spontaneous resistance emergence concerned with such targets might be rather low. On the other hand, resistance to PrAMPs may arise as a result of the inactivation of transporter proteins like SbmA or MdtM, which was shown for a number of relatively short peptides. Mammalian proline-rich cathelicidins usually have a length of >30 amino acid residues with the hydrophobic *C*-terminus and, therefore, typically are capable of effectively crossing the outer and cytoplasmic membranes of bacteria and even damage them at higher concentrations by switching to a membranolytic mechanism of action [7].

**Figure 4:**
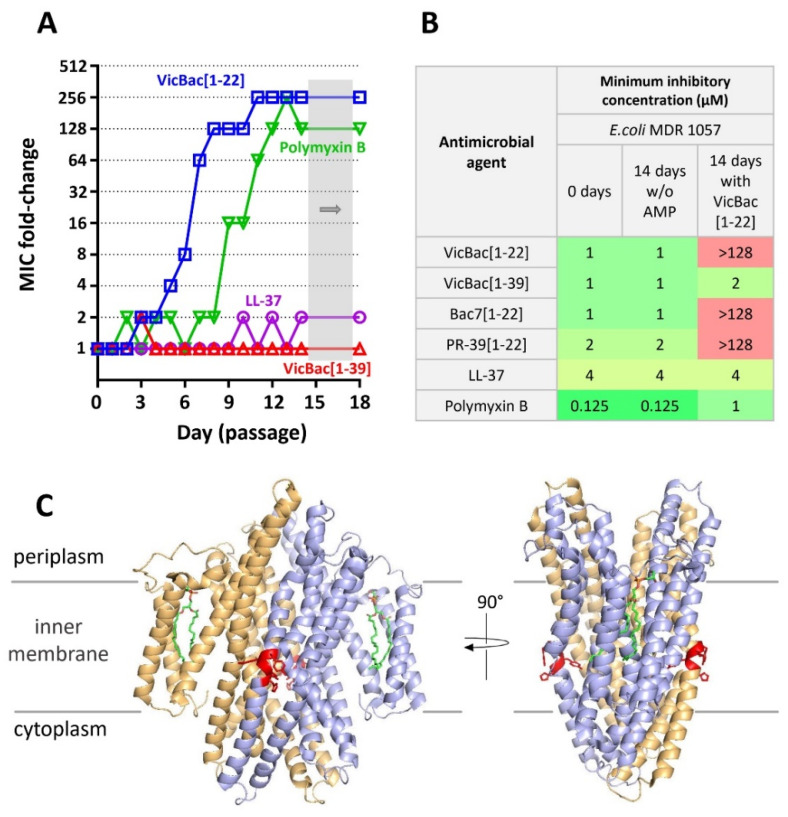
(**A**) Serial passage resistance induction by VicBac[1–39], its *N*-terminal fragment [1–22], the human cathelicidin LL-37, and polymyxin B against the sensitive clinical isolate *E. coli* MDR 1057. Bacteria that grew at the highest concentration of AMPs on the final day (the 14th day) were passaged further 3 times on drug-free agar plates before determining the final MIC value (gray shading). (**B**) Evaluation of cross-resistance effects. Antibacterial activity was determined in the rich Mueller-Hinton broth supplemented with 0.9% NaCl. (**C**) The localization of the tetrapeptide fragment His315-Tyr316-Met317-Tyr318 in the SbmA transporter is marked in red. The model of the SbmA transporter (PDB 7P34 [43]) was visualized with the PyMOL software. Two subunits of the SbmA homodimer are marked in different colors.

Here we used VicBac as a model molecule to prove this hypothesis by inducing experimental resistance to this peptide and its shortened analog VicBac[1–22]. The *E. coli* MDR 1057 strain was subjected to the resistance development test by subsequent culturing in the presence of VicBac, VicBac[1–22], the human cathelicidin LL-37, as well as antibiotic polymyxin B at increasing concentrations. The method used in this study allows for monitoring MIC values after each transfer. The 128-fold increase in MIC value was registered after 14-day selection by polymyxin B, which corresponds well to our previous results (Figure 4A). Expectedly, the MIC of membrane-targeting LL-37 increased only 2-fold. A 256-fold increase in MIC value (>128 μM) was registered after 10 passages subjected to selection by VicBac[1–22], and detectable MIC changes became visible after several initial transfers. Such resistance was stable, as a serial passage over three steps in the absence of the peptide did not change the MIC value. In contrast, the MIC of full-length VicBac did not change after the 2-week experiment, thus arguing the presence of the *C*-terminal hydrophobic motif prevented the formation of resistance against Pro-rich AMP.

### 3.5. Analysis of the Resistance Mechanisms to VicBac[1–22]

Then, the strain resistant to VicBac[1–22] was analyzed for cross-resistance to other antibacterial agents tested (Figure 4B). No differences in MICs before and after 14 passages without antimicrobial agents were observed (Figure 4B). The strong cross-resistance to short PrAMPs like Bac7[1–22] and PR-39[1–22] was found. Notably, we also did not observe any cross-resistance of the strain to the wild-type VicBac and control cathelicidin LL-37. In total, it may suggest that mechanisms of the resistance development to VicBac[1–22] in our experiment were associated with the modification of the membrane transporter system. To test this hypothesis, we performed whole-genome sequencing of VicBac[1–22]-resistant and referenced *E. coli* MDR 1057 strains. After the annotation of assembled contigs and the alignment on the reference genome, we identified an in-frame deletion of 12 base pairs in the *sbmA* gene of the resistant strain (Figure S5) that resulted in the deletion of the tetrapeptide fragment His315-Tyr316-Met317-Tyr318, which adopted the turn in transmembrane helix TM5 (Figure 4C). Interestingly, all fully resistant to different antibiotic variants of SbmA were shown to have single mutations in TM5, suggesting its important role in substrate translocation [43]. This deletion was also verified by the PCR-amplification of the *sbmA* gene, including its regulatory part in both strains, followed by Sanger sequencing.

Interestingly, inactivation of the *sbmA* gene led only to a 16-fold increase in MIC (from 1 to 16 µM, Figure 2B), while the VicBac[1–22]-resistant strain had the MIC of >128 µM. Therefore, two scenarios are possible: (1) the mutation allows SbmA to become a protection factor (for example, due to the peptide-binding); (2) the mutation leads to inactivation of SbmA and the additional increase in MIC is achieved due to any unspecified changes in bacterial cells. To discriminate between them, we checked the activity of the BW25113Δ*sbmA* strains having an additional plasmid-borne allele encoding either the wild-type SbmA or the mutant one bearing the tetrapeptide deletion. The target genes were expressed under the strong constitutive artificial promoter J23119 in the pUC vector (Figure S5). We found an 8-fold increase in MICs for the strain having a mutant phenotype compared with the wild-type SbmA (the observed increase in MICs from 1 to 8 µM). The control strain BW25113Δ*sbmA* expressing the control protein sfGFP under the same promoter had a MIC of 8 µM. This evidently indicates that the deletion inactivates the SbmA transporter. The whole-genome analysis did not reveal any additional mutations; however, other changes in the VicBac[1–22]-resistant strain are highly possible to achieve MIC of >128 µM. This is also supported by the cross-resistance effect to polymyxin B (from 0.125 to 1 µM, Figure 4B), which can be mediated by modifications of the LPS structure or the cell surface charge. Interestingly, the wild-type VicBac retains high activity against this resistant strain.

It is known that the SbmA transporter is a mutation-prone protein undergoing strong selective pressure when short PrAMPs are used to induce bacterial resistance *in vitro* [20,44]. As an activity of VicBac is practically independent of the expression of this transporter in *E. coli*, this peptide does not cause mutation in the gene encoding SbmA. To prevent transporter-independent translocation of the peptide, a significant change in membrane or cell surface structure must occur. On the other hand, both VicBac and VicBac[1–22] kill bacteria, predominantly inhibiting the translation process. Multiple contacts between PrAMPs and the ribosome minimize the risk of bacterial resistance development. Indeed, we did not find any mutations in ribosomal genes in the obtained VicBac[1–22]-resistant strain. Both the cytoplasmic membrane and the ribosome are highly conservative targets and their modifications will require a high fitness cost. Therefore, we can conclude that the presence of the *C*-terminal hydrophobic motif prevents bacterial resistance to VicBac due to its independence of penetration via the mutation-prone SbmA transporter.

The resistance to short PrAMPs can be overcome using a combination of them with pore-forming cationic AMPs [20]. Moreover, the resistance to PrAMPs that inhibit protein biosynthesis (for example, PR-39) leads to an increased sensitivity to pore-forming AMPs and vice versa [45]. Therefore, the short *N*-terminal fragment VicBac[1–22] can also be considered a potential drug candidate.

### 3.6. The Presence of the C-Terminal Part Does Not Provide Specific Immune-Modulatory Effects of the Cathelicidin VicBac but Enhances Them

**Figure 5:**
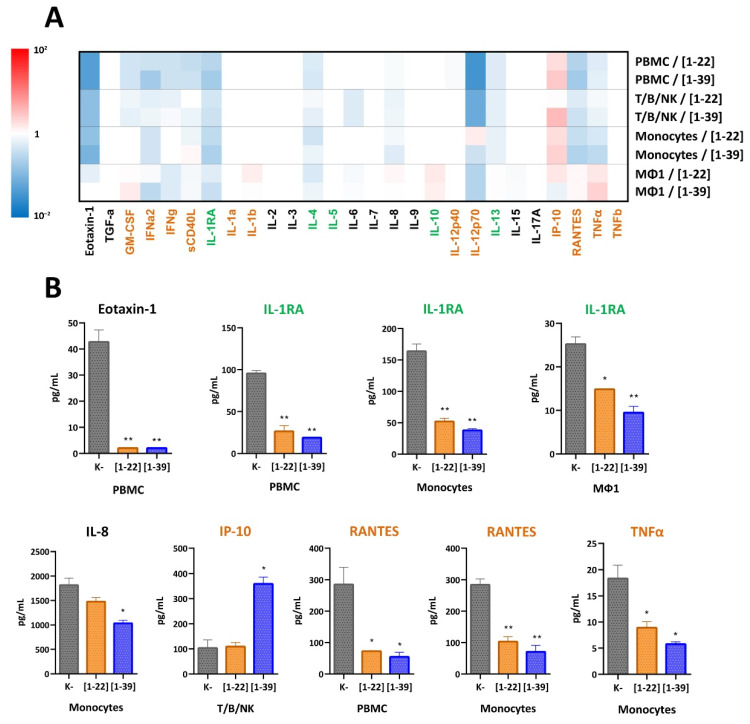
(**A**) Heat map representing profiles of cytokines/chemokines/growth factors production by different cell lines in response to incubation with 2 µM of the alpaca cathelicidin VicBac[1–39] and its *N*-terminal fragment [1–22] related to control without the peptide treatment. Proinflammatory factors are marked in orange; anti-inflammatory factors are marked in green. (**B**) Absolute levels of the cytokines and chemokines produced by different cell lines. Error bars represent the standard deviation (±SD) between two biological replications. Significance levels are: * *p* < 0.05, ** *p* < 0.01.

Due to the low cytotoxicity of PrAMPs, the study of naturally occurring peptides can be used to identify those ones that carry properties desirable in new immunomodulatory therapeutics, as well as to elucidate their functional role during infection. It has been previously shown that cathelicidins can induce both pro- and anti-inflammatory action by various cell types [46]. Moreover, the bovine proline-rich cathelicidin Bac5 was shown to act as a potentiator of the innate immune response in the animal model of Gram-negative bacterial infection [47]. Here, we aimed to find key structural elements of VicBac involved in immunomodulation (Figure 5A,B).

Earlier, the proline-rich peptide Bac7[1–35] accumulated selectively within the primed macrophages MΦ1 with respect to resting monocytes [48]. This may contribute to the overall difference observed in effects on MΦ1 and other tested cells after the VicBac treatment. In most cases, the inhibition of proinflammatory factors (cytokine TNFα and chemotactic IL-8/CXCL8, RANTES/CCL5, and eotaxin-1/CCL11) as well as anti-inflammatory IL-1RA was observed. Production of TNFα by monocytes was slightly inhibited from 18.47 to 10.31 pg/mL (*p* = 0.0418) by the *N*-terminal VicBac[1–22] and to 11.41 pg/mL (*p* = 0.020) by the full-length VicBac[1–39]. Production of IL-8/CXCL8, which is a key chemotactic cytokine involved in the recruitment of neutrophils to the site of damage or infection, was inhibited from 1813 to 1477 pg/mL (*p* = 0.010) by the peptide [1–22] and to 1034 pg/mL (*p* = 0.019) by the peptide [1–39]. Production of RANTES (Regulated on Activation, Normal T cell Expressed and Secreted), also known as the C-C motif chemokine 5 (CCL5), decreased on PBMC (from 286.63 in controls to 72.88 pg/mL, *p* = 0.0314 for the peptide [1–22] and to 54.72 pg/mL, *p* = 0.027 for the peptide [1–39]) and primary monocytes (from 284.28 to 103.33 pg/mL, *p* = 0.0089 for [1–22] and to 71.5 pg/mL, *p* = 0.0082 for [1–39]) upon incubation with the studied peptides. Production of the proinflammatory eosinophil chemotactic protein-1 (eotaxin-1/CCL11), which selectively recruits eosinophils to the site of pathogen invasion and is a key regulator of intestinal inflammation, by blood lymphocytes was also decreased from 42.69 to below the detectable minimum of <2.04 pg/mL, *p* < 0.0064. Decreasing of the anti-inflammatory IL-1RA, which is soluble interleukin-1 receptor antagonist, was observed on PBMC (from 95.52 to 26.39 pg/mL, *p* = 0.0059 for the peptide [1–22], and to 18.79 pg/mL, *p* = 0.0010 for the peptide [1–39]), pro-inflammatory macrophages (from 25.21 to 14.84 pg/mL, *p* = 0.0127 for the peptide [1–22], and to 9.47 pg/mL, *p* = 0.0098 for the peptide [1–39]), and primary monocytes (from 163.77 to 51.84 pg/mL, *p* = 0.0062 for the peptide [1–22], and to 37.82 pg/mL, *p* = 0.0043 for the peptide [1–39]). The only one analyte shown to be increased upon incubation of most of the cell lines with the studied peptides was the C-X-C motif chemokine ligand 10 (CXCL10), also known as the interferon-γ-induced protein 10 (IP-10). Interestingly, its production was elevated in the T/B/NK culture only in the case of the full-length cathelicidin [1–39], while the peptide [1–22] failed to induce its production. It has been previously shown that CXCL10/IP-10 expression, which attracts macrophages, T cells, NK cells, and DCs, was induced by LL-37, the mouse mCRAMP, the canine K9CATH, and the equine eCATH-3 [49].

Taken together, the data obtained report pro- and anti-inflammatory action of VicBac on various cell types. Interestingly, almost all analytes (both pro- and anti-inflammatory) were down-regulated by the peptides. This is in contrast to human LL-37, which caused more complicated immunomodulatory effects on different mammalian cells [46]. The immunomodulatory action of VicBac is mainly connected with the inhibition of proinflammatory TNFα and chemotactic cytokines IL-8/CXCL8, RANTES/CCL5, and eotaxin-1/CCL11, as well as anti-inflammatory IL-1RA. Moreover, both shortened [1–22] and full-length VicBac[1–39] cathelicidin variants have similar immunomodulatory action (both stimulation and inhibition) on the same cell types, with the latter peptide having more pronounced effects. Therefore, the core element of the peptide required for displaying this activity lies in the *N*-terminal part. This is similar to the results obtained with PR-39 and its analogs which inducted the TNFα production by porcine macrophages [50]. We also found stimulation of the TNFα production by macrophages (from 12.94 to 20.63 pg/mL for the peptide [1–22] and to 28.68 pg/mL for the peptide [1–39]), but a significance level was quite low in both cases due to high variability between biological replications. Apparently, the *C*-terminal region of VicBac plays an important but not key specific role in immune modulation. The only exception was found in the case of induction of the proinflammatory chemokine CXCL10/IP-10 increased in T/B/NK cells only upon incubation with the full-length VicBac[1–39].

## 4. Conclusions

Search and design of new PrAMPs combining a short peptide “war-head” inhibiting 70S ribosome or other intracellular targets like bacterial chaperon DnaK and an effective membrane permeabilization tag avoiding penetration via mutation-prone transporters like SbmA is a promising approach to develop novel peptide antibiotics. In this study, we identified a novel family of proline-rich cathelicidins from *Camelidae* mammalians which resemble the porcine PR-39 peptide with a quite similar mechanism of action, peptide length, and amino acid composition but have comparatively low sequence homology. Previously, using the Bac7 [39] or OaBac7.5mini [51] peptides, it was shown that the *C*-terminal part of the peptide itself did not display antibacterial activity, which indicated its auxiliary function. Indeed, the key ribosome-targeting structural element of Bac7 is the fragment [1–16], while the presence of the *C*-terminal region greatly expanded the spectrum of antibacterial activity but, at the same time, increased the cytotoxicity of the peptide. Similar results were obtained with the C12-lipidated Bac7[1–16] analog, named Bac-C12 [52]. Moreover, such a lipidation extended the activity against different *S. aureus* strains and prevented the selection of resistant bacteria in vitro. In total, our results in the structure-functional study of the novel proline-rich cathelicidin VicBac from the alpaca *V. pacos* supported these data. Recent studies on PrAMPs have shifted an initial focus from studying antibacterial activity to elucidating their potential roles as immunomodulators. Our results showed that the *N*-terminal part [1–22] of VicBac was sufficient for the antimicrobial activity and the strong inhibition of bacterial translation, as well as for the stimulation of most found immune response effects by human immune cells in vitro. The presence of the *C*-terminal tail in the natural VicBac does not provide specific immune-modulatory effects but enhances the observed impact as compared with the truncated analog. Moreover, a quite hydrophobic extended *C*-terminal part of VicBac allows this peptide to penetrate/damage biological membranes and prevent bacterial resistance effectively. The marked antibacterial activity of VicBac, along with its moderate adverse effects on mammalian cells, make this molecule a promising scaffold for the development of a novel peptide antibiotic.

## Data Availability

All data generated and analyzed during this study are included in this published article and its Supplementary Materials file.

## Supplementary Materials

The following supporting information can be downloaded at: https://www.mdpi.com/article/10.3390/membranes12050515/s1, Figure S1: Reverse-phase high-performance liquid chromatography (RP-HPLC) purification of the recombinant VicBac[1–22]; Figure S2: (A) Plasmid vector for the expression of sfGFP under the strong constitutive artificial promoter J23119. (B) The scheme of DNA-amplification, ligase-independent assembly of the complementation plasmids overexpressing SbmA variants in SbmA-deficient *E. coli*; Figure S3: Helical wheel projections of alpaca cathelicidins; Figure S4: Structure of *CATHL* genes and corresponding preprocathelicidins of *Vicugna pacos*; Figure S5: Alignment of nucleotide sequences of genes encoding SbmA from *E. coli* MDR CI 1057 “wt” (wild type) and mutant SbmA from VicBac[1–22]-resistant *E. coli* MDR CI 1057 “mut” obtained after selection; Table S1: Amino acid sequences and molecular masses of the peptides used in this study; Table S2: Amino acid sequences of natural cathelicidins from *Camelidae* species.

## References

[1] Munir M.U., Ahmed A., Usman M., Salman S. (2020). Recent Advances in Nanotechnology-Aided Materials in Combating Microbial Resistance and Functioning as Antibiotics Substitutes. Int. J. Nanomed..

[2] Munir M.U., Ahmad M.M. (2022). Nanomaterials Aiming to Tackle Antibiotic-Resistant Bacteria. Pharmaceutics.

[3] Laxminarayan R., Duse A., Wattal C., Zaidi A.K.M., Wertheim H.F.L., Sumpradit N., Vlieghe E., Hara G.L., Gould I.M., Goossens H. (2013). Antibiotic Resistance—the Need for Global Solutions. Lancet Infect. Dis..

[4] Graf M., Mardirossian M., Nguyen F., Seefeldt A.C., Guichard G., Scocchi M., Innis C.A., Wilson D.N. (2017). Proline-Rich Antimicrobial Peptides Targeting Protein Synthesis. Nat. Prod. Rep..

[5] Kopeikin P.M., Zharkova M.S., Kolobov A.A., Smirnova M.P., Sukhareva M.S., Umnyakova E.S., Kokryakov V.N., Orlov D.S., Milman B.L., Balandin S.V. (2020). Caprine Bactenecins as Promising Tools for Developing New Antimicrobial and Antitumor Drugs. Front. Cell. Infect. Microbiol..

[6] Seefeldt A.C., Graf M., Pérébaskine N., Nguyen F., Arenz S., Mardirossian M., Scocchi M., Wilson D.N., Innis C.A. (2016). Structure of the Mammalian Antimicrobial Peptide Bac7 (1–16) Bound within the Exit Tunnel of a Bacterial Ribosome. Nucleic Acids Res..

[7] Mardirossian M., Pérébaskine N., Benincasa M., Gambato S., Hofmann S., Huter P., Müller C., Hilpert K., Innis C.A., Tossi A. (2018). The Dolphin Proline-Rich Antimicrobial Peptide Tur1A Inhibits Protein Synthesis by Targeting the Bacterial Ribosome. Cell Chem. Biol..

[8] Florin T., Maracci C., Graf M., Karki P., Klepacki D., Berninghausen O., Beckmann R., Vázquez-Laslop N., Wilson D.N., Rodnina M.V. (2017). An Antimicrobial Peptide That Inhibits Translation by Trapping Release Factors on the Ribosome. Nat. Struct. Mol. Biol..

[9] Gagnon M.G., Roy R.N., Lomakin I.B., Florin T., Mankin A.S., Steitz T.A. (2016). Structures of Proline-Rich Peptides Bound to the Ribosome Reveal a Common Mechanism of Protein Synthesis Inhibition. Nucleic Acids Res..

[10] Lázár V., Martins A., Spohn R., Daruka L., Grézal G., Fekete G., Számel M., Jangir P.K., Kintses B., Csörgő B. (2018). Antibiotic-Resistant Bacteria Show Widespread Collateral Sensitivity to Antimicrobial Peptides. Nat. Microbiol..

[11] Holfeld L., Herth N., Singer D., Hoffmann R., Knappe D. (2015). Immunogenicity and Pharmacokinetics of Short, Proline-Rich Antimicrobial Peptides. Future Med. Chem..

[12] Shinnar A.E., Butler K.L., Park H.J. (2003). Cathelicidin Family of Antimicrobial Peptides: Proteolytic Processing and Protease Resistance. Bioorgan. Chem..

[13] Holfeld L., Knappe D., Hoffmann R. (2018). Proline-Rich Antimicrobial Peptides Show a Long-Lasting Post-Antibiotic Effect on Enterobacteriaceae and Pseudomonas Aeruginosa. J. Antimicrob. Chemother..

[14] Lai P.-K., Geldart K., Ritter S., Kaznessis Y.N., Hackel B.J. (2018). Systematic Mutagenesis of Oncocin Reveals Enhanced Activity and Insights into the Mechanisms of Antimicrobial Activity. Mol. Syst. Des. Eng..

[15] Seefeldt A.C., Nguyen F., Antunes S., Pérébaskine N., Graf M., Arenz S., Inampudi K.K., Douat C., Guichard G., Wilson D.N. (2015). The Proline-Rich Antimicrobial Peptide Onc112 Inhibits Translation by Blocking and Destabilizing the Initiation Complex. Nat. Struct. Mol. Biol..

[16] Chernysh S., Gordya N., Suborova T. (2015). Insect Antimicrobial Peptide Complexes Prevent Resistance Development in Bacteria. PLoS ONE.

[17] Mattiuzzo M., Bandiera A., Gennaro R., Benincasa M., Pacor S., Antcheva N., Scocchi M. (2007). Role of the Escherichia Coli SbmA in the Antimicrobial Activity of Proline-Rich Peptides. Mol. Microbiol..

[18] Krizsan A., Knappe D., Hoffmann R. (2015). Influence of the *YjiL-MdtM* Gene Cluster on the Antibacterial Activity of Proline-Rich Antimicrobial Peptides Overcoming Escherichia Coli Resistance Induced by the Missing SbmA Transporter System. Antimicrob. Agents Chemother..

[19] Bolosov I.A., Panteleev P.V., Sychev S.V., Sukhanov S.V., Mironov P.A., Myshkin M.Y., Shenkarev Z.O., Ovchinnikova T.V. (2021). Dodecapeptide Cathelicidins of Cetartiodactyla: Structure, Mechanism of Antimicrobial Action, and Synergistic Interaction With Other Cathelicidins. Front. Microbiol..

[20] Panteleev P.V., Bolosov I.A., Kalashnikov A.À., Kokryakov V.N., Shamova O.V., Emelianova A.A., Balandin S.V., Ovchinnikova T.V. (2018). Combined Antibacterial Effects of Goat Cathelicidins With Different Mechanisms of Action. Front. Microbiol..

[21] Sola R., Mardirossian M., Beckert B., Sanghez De Luna L., Prickett D., Tossi A., Wilson D.N., Scocchi M. (2020). Characterization of Cetacean Proline-Rich Antimicrobial Peptides Displaying Activity against ESKAPE Pathogens. Int. J. Mol. Sci..

[22] Hassanin A., Delsuc F., Ropiquet A., Hammer C., Jansen van Vuuren B., Matthee C., Ruiz-Garcia M., Catzeflis F., Areskoug V., Nguyen T.T. (2012). Pattern and Timing of Diversification of Cetartiodactyla (Mammalia, Laurasiatheria), as Revealed by a Comprehensive Analysis of Mitochondrial Genomes. C. R. Biol..

[23] Kulkarni S.S., Falzarano D. (2021). Unique Aspects of Adaptive Immunity in Camelids and Their Applications. Mol. Immunol..

[24] Studier F.W. (2005). Protein Production by Auto-Induction in High Density Shaking Cultures. Protein Expr. Purif..

[25] Baba T., Ara T., Hasegawa M., Takai Y., Okumura Y., Baba M., Datsenko K.A., Tomita M., Wanner B.L., Mori H. (2006). Construction of *Escherichia Coli* K-12 In-frame, Single-gene Knockout Mutants: The Keio Collection. Mol. Syst. Biol..

[26] de Sena Brandine G., Smith A.D. (2021). Falco: High-Speed FastQC Emulation for Quality Control of Sequencing Data. F1000Research.

[27] Bolger A.M., Lohse M., Usadel B. (2014). Trimmomatic: A Flexible Trimmer for Illumina Sequence Data. Bioinformatics.

[28] Bankevich A., Nurk S., Antipov D., Gurevich A.A., Dvorkin M., Kulikov A.S., Lesin V.M., Nikolenko S.I., Pham S., Prjibelski A.D. (2012). SPAdes: A New Genome Assembly Algorithm and Its Applications to Single-Cell Sequencing. J. Comput. Biol..

[29] Gurevich A., Saveliev V., Vyahhi N., Tesler G. (2013). QUAST: Quality Assessment Tool for Genome Assemblies. Bioinformatics.

[30] Seemann T. (2014). Prokka: Rapid Prokaryotic Genome Annotation. Bioinformatics.

[31] Li H., Durbin R. (2009). Fast and Accurate Short Read Alignment with Burrows-Wheeler Transform. Bioinformatics.

[32] Koboldt D.C., Zhang Q., Larson D.E., Shen D., McLellan M.D., Lin L., Miller C.A., Mardis E.R., Ding L., Wilson R.K. (2012). VarScan 2: Somatic Mutation and Copy Number Alteration Discovery in Cancer by Exome Sequencing. Genome Res..

[33] Yan Q., Fong S.S. (2017). Study of in Vitro Transcriptional Binding Effects and Noise Using Constitutive Promoters Combined with UP Element Sequences in Escherichia Coli. J. Biol. Eng..

[34] Beyer H.M., Gonschorek P., Samodelov S.L., Meier M., Weber W., Zurbriggen M.D. (2015). AQUA Cloning: A Versatile and Simple Enzyme-Free Cloning Approach. PLoS ONE.

[35] Panteleev P.V., Bolosov I.A., Ovchinnikova T.V. (2016). Bioengineering and Functional Characterization of Arenicin Shortened Analogs with Enhanced Antibacterial Activity and Cell Selectivity: Bioengineering of Arenicin Shortened Analogs with Enhanced Selectivity. J. Pept. Sci..

[36] Genin M., Clement F., Fattaccioli A., Raes M., Michiels C. (2015). M1 and M2 Macrophages Derived from THP-1 Cells Differentially Modulate the Response of Cancer Cells to Etoposide. BMC Cancer.

[37] Whelehan C.J., Barry-Reidy A., Meade K.G., Eckersall P., Chapwanya A., Narciandi F., Lloyd A.T., O’Farrelly C. (2014). Characterisation and Expression Profile of the Bovine Cathelicidin Gene Repertoire in Mammary Tissue. BMC Genom..

[38] Mardirossian M., Sola R., Beckert B., Valencic E., Collis D.W.P., Borišek J., Armas F., Di Stasi A., Buchmann J., Syroegin E.A. (2020). Peptide Inhibitors of Bacterial Protein Synthesis with Broad Spectrum and SbmA-Independent Bactericidal Activity against Clinical Pathogens. J. Med. Chem..

[39] Benincasa M., Scocchi M., Podda E., Skerlavaj B., Dolzani L., Gennaro R. (2004). Antimicrobial Activity of Bac7 Fragments against Drug-Resistant Clinical Isolates. Peptides.

[40] Metelev M., Osterman I.A., Ghilarov D., Khabibullina N.F., Yakimov A., Shabalin K., Utkina I., Travin D.Y., Komarova E.S., Serebryakova M. (2017). Klebsazolicin Inhibits 70S Ribosome by Obstructing the Peptide Exit Tunnel. Nat. Chem. Biol..

[41] Lazzaro B.P., Zasloff M., Rolff J. (2020). Antimicrobial Peptides: Application Informed by Evolution. Science.

[42] Brakel A., Krizsan A., Itzenga R., Kraus C.N., Otvos L., Hoffmann R. (2022). Influence of Substitutions in the Binding Motif of Proline-Rich Antimicrobial Peptide ARV-1502 on 70S Ribosome Binding and Antimicrobial Activity. Int. J. Mol. Sci..

[43] Ghilarov D., Inaba-Inoue S., Stepien P., Qu F., Michalczyk E., Pakosz Z., Nomura N., Ogasawara S., Walker G.C., Rebuffat S. (2021). Molecular Mechanism of SbmA, a Promiscuous Transporter Exploited by Antimicrobial Peptides. Sci. Adv..

[44] Schmidt R., Krizsan A., Volke D., Knappe D., Hoffmann R. (2016). Identification of New Resistance Mechanisms in *Escherichia Coli* against Apidaecin 1b Using Quantitative Gel- and LC–MS-Based Proteomics. J. Proteome Res..

[45] Spohn R., Daruka L., Lázár V., Martins A., Vidovics F., Grézal G., Méhi O., Kintses B., Számel M., Jangir P.K. (2019). Integrated Evolutionary Analysis Reveals Antimicrobial Peptides with Limited Resistance. Nat. Commun..

[46] van Harten R., van Woudenbergh E., van Dijk A., Haagsman H. (2018). Cathelicidins: Immunomodulatory Antimicrobials. Vaccines.

[47] Price R.L., Bugeon L., Mostowy S., Makendi C., Wren B.W., Williams H.D., Willcocks S.J. (2019). In Vitro and in Vivo Properties of the Bovine Antimicrobial Peptide, Bactenecin 5. PLoS ONE.

[48] Pelillo C., Benincasa M., Scocchi M., Gennaro R., Tossi A., Pacor S. (2014). Cellular Internalization and Cytotoxicity of the Antimicrobial Proline-Rich Peptide Bac7 (1–35) in Monocytes/Macrophages, and Its Activity Against Phagocytosed Salmonella Typhimurium. Protein Pept. Lett..

[49] Coorens M., Scheenstra M.R., Veldhuizen E.J.A., Haagsman H.P. (2017). Interspecies Cathelicidin Comparison Reveals Divergence in Antimicrobial Activity, TLR Modulation, Chemokine Induction and Regulation of Phagocytosis. Sci. Rep..

[50] Veldhuizen E.J.A., Schneider V.A.F., Agustiandari H., van Dijk A., Tjeerdsma-van Bokhoven J.L.M., Bikker F.J., Haagsman H.P. (2014). Antimicrobial and Immunomodulatory Activities of PR-39 Derived Peptides. PLoS ONE.

[51] Anderson R.C., Hancock R.E.W., Yu P.-L. (2004). Antimicrobial Activity and Bacterial-Membrane Interaction of Ovine-Derived Cathelicidins. Antimicrob. Agents Chemother..

[52] Armas F., Di Stasi A., Mardirossian M., Romani A.A., Benincasa M., Scocchi M. (2021). Effects of Lipidation on a Proline-Rich Antibacterial Peptide. Int. J. Mol. Sci..

