# Supplementary material for: A Novel Proline-Rich Cathelicidin from the Alpaca Vicugna pacos with Potency to Combat Antibiotic-Resistant Bacteria: Mechanism of Action and the Functional Role of the C-Terminal Region
Source: Membranes (Basel). 2022 May 12;12(5):515. doi: 10.3390/membranes12050515 (PMC9146984; doi:10.3390/membranes12050515)
Supplement: Supplementary file 1 [file membranes-12-00515-s001.zip › membranes-1707256-supplementary.pdf]

Supplementary Materials

A novel proline-rich cathelicidin from the alpaca *Vicugna pacos* with potency to combat antibiotic-resistant bacteria: mechanism of action and the functional role of the *C*-terminal region

Pavel V. Panteleev *, Victoria N. Safronova, Roman N. Kruglikov, Ilia A. Bolosov, Ivan V. Bogdanov, Tatiana V. Ovchinnikova

M.M. Shemyakin & Yu.A. Ovchinnikov Institute of Bioorganic Chemistry, the Russian Academy of Sciences, Miklukho-Maklaya str., 16/10, 117997 Moscow, Russia; victoria.saf@ibch.ru (V.N.S.); kruglikov1911@mail.ru (R.N.K.); bolosov@ibch.ru (I.A.B.); contraton@mail.ru (I.V.B.); ovch@ibch.ru (T.V.O.)

* Correspondence: ibch@inbox.ru; Tel.: +7-495-335-09-00

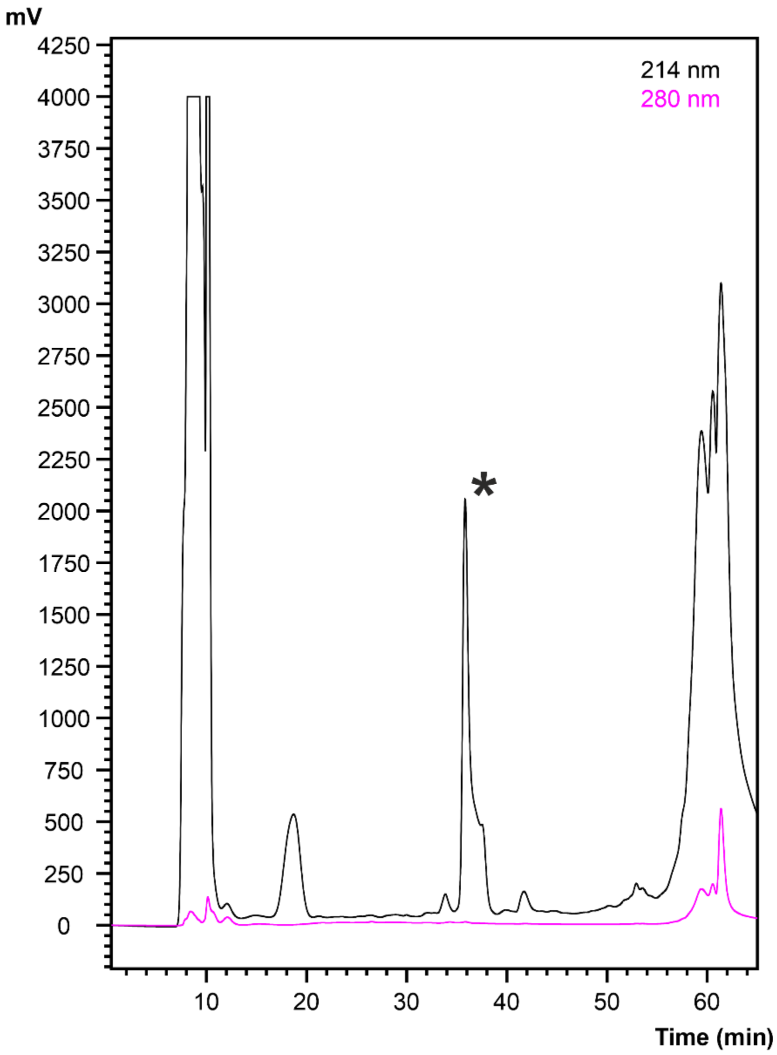

Figure S1. Reverse-phase high-performance liquid chromatography (RP-HPLC) purification of the recombinant VicBac[1-22]. The fraction of the target peptide is marked with an asterisk.

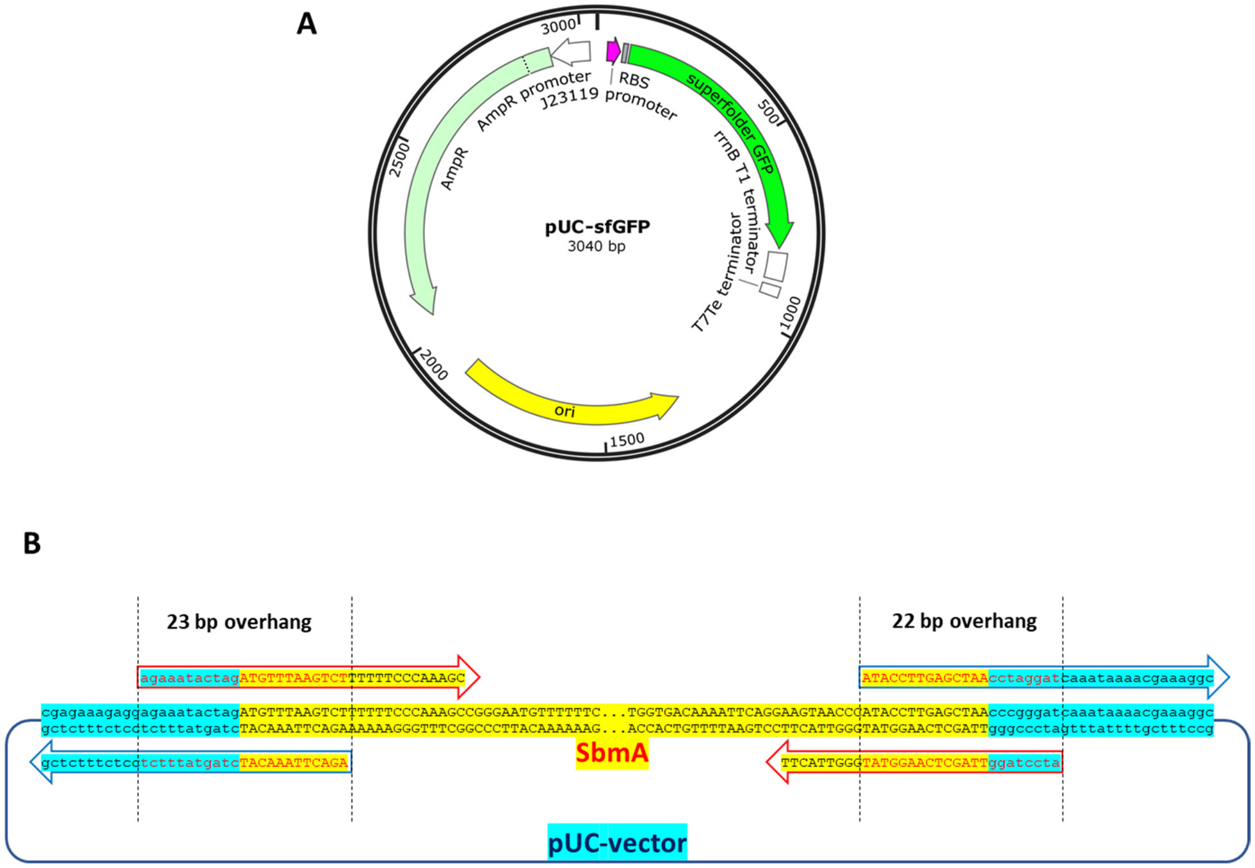

Figure S2. (A) Plasmid vector for the expression of sfGFP under the strong constitutive artificial promoter J23119. Map was visualized with SnapGene software. **(B)** The scheme of DNA-amplification, ligase-independent assembly of the complementation plasmids overexpressing SbmA variants in SbmA-deficient *E. coli*.

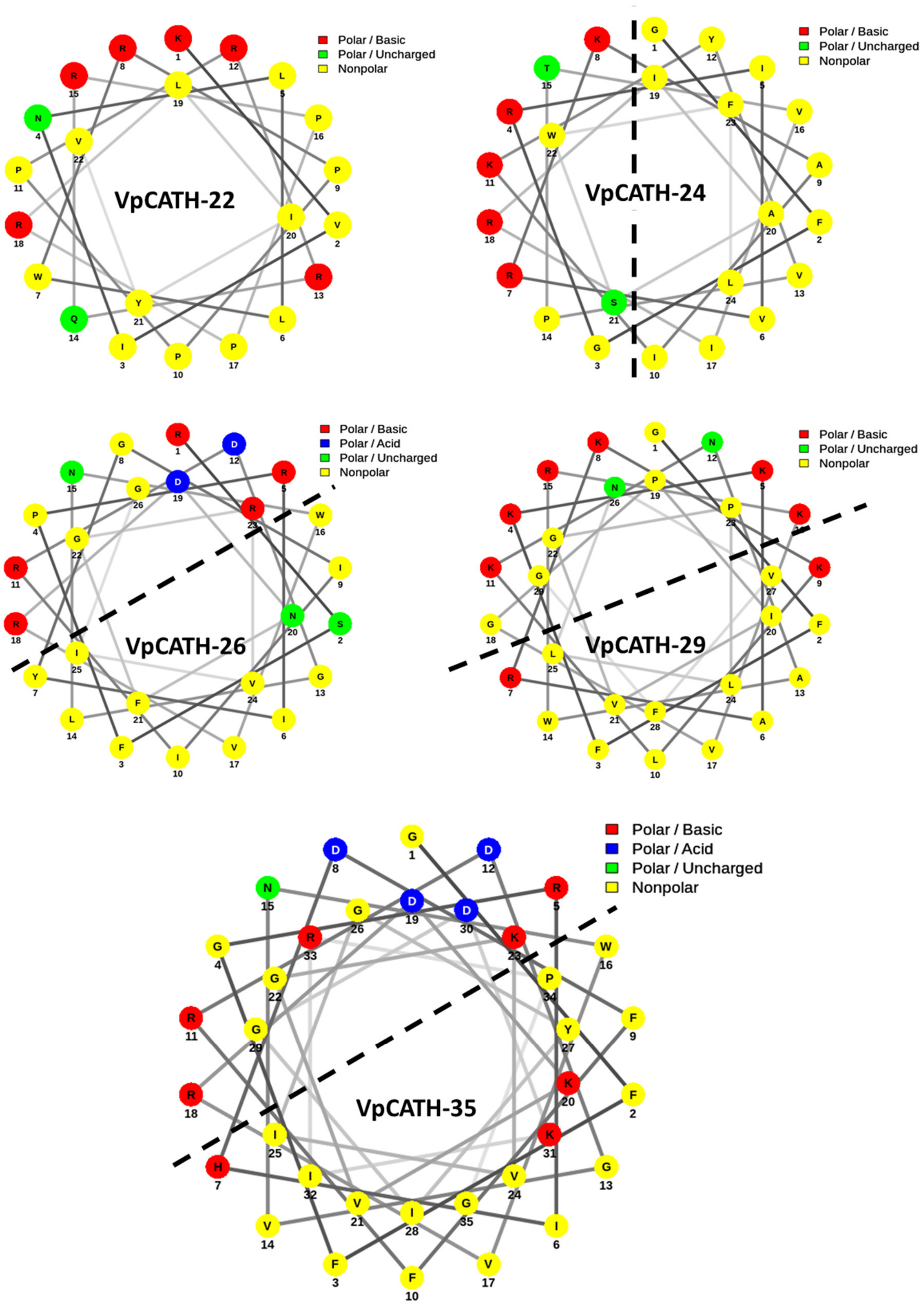

Figure S3. Helical wheel projections of alpaca cathelicidins. The projections were generated by online NetWheels tool (http://lbqp.unb.br/NetWheels/).

Vicugna pacos (GenBank: JEMW01023459.1)

METQRASLSLRRWSLWLLLLGLAVPWASAQALSYREAVLRAVDRLNEQSSDPNLYRLLELDLPPKADEDPDAPKPVSFTVKETVCPRRTQLPQEQCA
FKENGVVKQCLGTVNLYQLRDNFDITCNELQSV**KVINLLWRPPPRRQRPPRLIYV** (VpCATH-22)

cactgcccataacacaccccatggggcccaggagaggccaagaattttcagggagttggagaaaccatttcttcaccttccacaggggcagacctc
acccacagccagcaatcccctgaggcaagagcggggcctgaggcaactccagctcacaccctggcctgggacagggatggggcaggtggggaaggct
cctgcttgagcaattgcatcaggcccaggctgagc**ataaa**ggagggtcctgtgggctgggacgaggcagactcggggaccatggagacccagagggc
cagcctctccctgcgccgctggtcactgtggctactgctgctgggactagcggtgccttgggccagcgcccaggccctgagctatagggaggcggtg
cttcgagctgtggatcgcctcaatgagcagtcctcagaccccaatctctaccgcctcctggagctggacctgccgcccaaggctgtgagtcggggag
ggtctggggagggaggcgtctcccgccaggcttggccacactgtccctccctttgctcgcgctgtacctcctgtcaggaaggcacctctccctctaa
gtgggatcccacctctcacagaaatcctcccagagctgggtcccctcccgttgtgagagccttctgcctggcatctctgctgtgggaacagagccct
gacagcccgctcaggctccagggactcctgggagctccagggatacgctgggctcactggctctgggatgtgacttccctgcttccagtcccctgtg
caccccattgtctccccaccagggaagggctgtgctcagcctggggggttccagtgactaggtctttccctgcaggtgtcccctgacttccctcagcc
cctccaaggggagctgcccctcaggagagcgggtctgaggtcccctcctgttccctgtgcacccgggagctcaggatcaggcatatgggagggggctg
tcaaggggggccgtcctgctgggggggcagggagacaggtcatacaaggaaggatgagcctgagccagtctccccactttgatccttgagcaggacgag
gacccagacgccccgaagcctgtgagcttcacggtgaaggagaccgtgtgtcccaggaggacacagctgccacaggagcagtgtgccttcaaggaga
atggggtgaggctgggggcttagggcgctggtgggttcctcccaaggacccgaacaggaggcttggggcaggtggtggttagcagcccctggggtga
ggctgggtgtttatggctcaggggttccagtgtgacctggagtctccccttccaggtggtgaaacagtgtttggggacagtcaacctgtaccagctc
agggacaacttcgatataacatgtaatgaggtgagtgacccccttctgggcttcagaggctgctggtggagatggtgtgtggaacagcctttgggcca
attcccctctgtgccatccctgtagccgcaggaaagccctcctcccctggctccccctcacccgatccccaggtctccaggccgggctctgcaatcc
cttagagcagtggttctctcagtgggctccccacttgggaactagagaaaaaggctgattctcaggcctgaccagacctacggaatcactgtgtgag
gtggagcccagtaatttctatttaagagcccttctggggggttttctgagtgtgctgaagcttgagaggcagtgactagagacaggacctttctgcc
cccgctcccgtccagcttttctgagatgggtttgtgcccctggaagccccctgtcatctctggaccttggtcctcatactgtctgtggctgtaatga
ttcaaccacgtgctccaaaggtcacagccagagaagtggtgctcttgacctcctgggtggaccatggaaaatcgcagtgcaggttgggaaggacctt
atgttgatcttggttcattaataaaaacaaacctctttacacttggttcacagctccagagtgtcaaggtcattaaccttctatggagaccaccacc
acggcggcagagacctcctagactgatttatgtatagttcagagattgggttgattatcagattatcagcatatcaaggattttctttaatcttga
ggtagcgaagatcctaaagatcagcttgtttcctctcaggctttggggctgtgaaa*aataa*attcttctgaaaacagcttcatcaggttgctcccac
ttactgtgaccaagtccttaattctgggaggctgagtgtgtgggcgggtgtgtgtgcacatgagcatgtgtgtgtgtgtctgtgtgtgc

Vicugna pacos (JEMW01020494.1)

METQRASLSLRCWSLWLLLLGLAVPWASAQALSYREAVLRAVDRLNEQSSDPNLYRLLELDLPPKADENPDAPKPVSFMVKETVCPRRTQQPPEQCD
FKENGVVKQCLGIVTLDQITGQLAVTCDELQSV**GFGRIVRKAIKYVPTVIRIASWFL** (VpCATH-24)

gtagggcagagcctggtcactccatctcctggagtattcaaccctctggagcaaccctgcccatgaaactccccatggggcccaggagaggccaaga
tgttgtcagggagatggagaaaccatttcttcaccttgcacaggaggcagacctcacccactgcccagcaatcccctgaggcaagagcggggcctga
ggcaactccagctcacaccctggccgggggcagggatggggcaggtggggaaggatcctgcttgaggaattgcatcaggcccaggctgagc**ataaa**g
gagggtcctgtgggctgggacgaggctgactcggggaccatggagacccagagggccagcctctccctgcgctgctggtcactgtggctactgctgc
tgggactagcggtgccttgggccagcgcccaggccctgagctatagggaggcggtgcttcgagctgtggatcgcctcaatgagcagtcctcagaccc
caatctctaccgcctcctggagctggacctgccgcccaaggctgtgagtcggggaggggctggggagggaggcgtctcccgccaggcttggccacac
tgtccctccctttgctcgcgctgtacctcctgtcaggaaggcacctctccctctaagtgggatcccacctctcacagaaatcctcccagagctgggt
cccctcctgttgtgagagccttctgcctggcatctctgctgtgggaacagagccctgacagcccgctcaggctccagggcctcctgggggctccagg
gcctcctgggggctccagggatacgctgggctcactggctctgggatgtgacttccctgcttccagtcccctgtgcaccccattgtctccccaccag
ggaagggctgtgctcagcctggggggttccagtgacaaggtctttccctgcaggtgtcccctgacttccctcagcccctccaaggggagctgcccctc
aggactcagggtctgaggtccccttctgctccctgtgtgcctgggagcccaggaccaggctgtgtcctcctcccctctgcacccagcccccagccca
gggccaggcacatgggagggggctgtcaaggggggccgtcctgccggggggcagggagaaaggtcatcaaggaagcatcagccttagccagtctcccca
ctttgatccttgagcaggacgagaacccagacgccccgaagcctgtgagcttcatggtgaaggagaccgtgtgtcccaggaggacacagcagccacc
ggagcagtgtgacttcaaggagaatggggtgaggttgggagctaggagcactggtgggtgcctccccaggaccctaacagaaggcttcagggagtgg
ttagcagctcctggggtgaggctgggtgtttatggctcaggggttccagtctgacctggagtctccccttccaggtggtgaaacagtgtttggggat
agtcaccctggaccagatcacaggccaattggcagtcacctgtgatgaggtgagtgacccccttctgggctgcaagggctgctggtgggggtggtgtg
tggaatattctttggaccaattaccttacgtcccatccagggcagggcaagtcctggaccccgctcactggagccccagatctcccactcaggctct
ggaatcccttagagcagtggttctctaagtgggcttcccacccagggcttgtcagaaaagcagattctcaggccccactcatacctactgaattaga
gtctgtggtggggctcagcagtttgtattttaagaagtcgtcctgaggattctgtgctgaagtttaagaggcattgagcacagtaaaaggctttcaa
cccctgcttctgtcaagctttgctgggatgggcttgtgaccctgggaagccccttgtcatctctggaccttggtttcctcatccgtgtgtgggtgta
gggattcaaccatatgctccaaaagtcacagccaaagggtgaactggggacacaaggcccctggggtcacccaggtaggggaggtgtgtaaatggga
aagattctgttcttacatctgggttcatttctcacaagtgacctgtttcttcccatgtacagctccagagtgtcggttttggcaggatagtaagaaa
ggcgatcaagtatgtcccgacagtcatccgtatagccagctggtttttgtgaattgtgagtccaggaaagagtcctaagggtccacctgtcttggct
caggttactagactctgaga*aataa*attctggtgaaaacaacctcctccaggcttcaatttcacttttctcccctcctcccacttaccaagattgca
tccttaactctgggaggccttgtgatatgcgtgtgtgtgtgtgtgtgtctgt

Figure S4 (part 1). Structure of *CATHL* genes and corresponding preprocathelicidins of *Vicugna pacos*. Predicted intron/exon boundaries are highlighted in red. Exons are highlighted in green. Signal peptide sequences are marked in yellow/black. Signal peptide sequence identified with SignalP 5.0 (www.cbs.dtu.dk/services/SignalP/). Peptide sequences of putative mature cathelicidins are marked with red font. The TATA-box signal is in bold and grey marked and the polyadenylation signal is in italics and grey marked.

Vicugna pacos (GenBank: ABRR03000981.1)

METQRDSLSLRRWSLWLLLLGLAVPWASAQALSYREAVLRAVDRLNEQSSDPNLYRLLELDLPPKADEDPDAPKPVSFTVKETVCPRRTQLPPQQCA
FKENGVVKQCLGTVNLYQLRDNFDITCNELQSV**RSFPRIYGIIRDGLNWVRDNFGRVIG** (VpCATH-26)

ggagaggccaagaatttcagggagatggagaaaccatttcttcaccttccacaggggcagacctcacccaccgcccagcaatcccctgaggcaag
agcggggcctgaggcaactccagctcacaccctggccggggacagggatggggcaggtggggaaggctcctgcttgagcaattgcatcaggcccagg
ctgagc**ataaa**ggagggtcctgtgggctgggacgaggccgactcggggaccatggagacccagagggacagcctctccctgcgccgctggtcactgt
ggctactgctgctgggactagcggtgccttgggccagcgcccaggccctgagctatagggaggcggtgcttcgagctgtggatcgcctcaatgagca
gtcctcagaccccaatctctaccgcctcctggagctggacctgccgcccaaggctgtgagtcggggagggactggggagggaggcgtctcccgccag
gcttggcctcgctgtccctccctttgctcgcgctgtacctcctgtcaggaaggcacctctccctctaagtgggatcccacctctcacagaaatcctc
ccagagctgggtcccctcccgttgtgagagccttctgcctggcatctctgctgtgggaacagagccctgacagctcgctcaggctccagggcctcct
gggggctccagggatacgctggggtcactggctctgggatgtgacttccctgcttccagtcccctatgcaccccattgtctccccaccagggaaggg
ctgtgctcagcctggggttccagtgacaaggtctttccttgcaggtatccgctgacttccctcagcccctccaagagggaggtgcccctcaggagag
cgggtgtgaggtcccctcctgctccctgtgaccctgggatcccaggaccaggctgtgtcctactcccctctgcacccagcccccagcccagtgccag
gaacatgggacaggctctcaaaggggtttgtccttctgggggagggagacaggtcacccaaggaagcatgagcctgagccagtctccccactttga
tccttgagcaggacgaggacccagacgccccgaagcctgtgagcttcacagtgaaggagaccgtgtgtcccaggaggacacagctgccaccgcagca
gtgtgccttcaaggagaatggggtgaggctgggggcttagggcgctggtgggtgcctcccaaggacccgaacaggaggtttggggtgggtggtggtt
agcagctcctggggtgaggagggtgtttatgcctcaggggttccagtgtgacctggagtctccccttccaggtggtgaaacagtgtttggggacag
tcaacctgtaccagctgagggacaacttcgatataacatgtaatgaggtgagtgaccccttgggctgcaggggctgctggtggaggtggtgtgtgga
acagcctttgggccaattcccctctgtgccatccctgtagccgcaggaaagccctcctcccctggctcccccctcacccgatccccaggtctcctggc
cgggctctgcaatcccttagagcagtggttctctcagtgggctccccacttgggaactagagaaaaaggctgattctcaggcctcattcagacctac
ggaagcattgtctgacttggagaccagtaatgtctattttaagagcccttctgggggatgctgactgtgctgaagcttgagtgcactgactagaata
atagactttctgcccctgctcccatccagcttttctgacatgggtttgtgcccctgggaagcccccctttcatctcaggaccttggtcctcatctgtc
tgtgtgagagatgattcaaccacgtgctccaagtgacacagacaaggaggtcaaatggtgcctcaagtctccttcggtggtccaagatggaagattt
ccaggtggggagggatctggtcttgtccctaagtcagtcctcacaagaatcctgtttctttctgctgcacagctccaaagcgtccggtcatttcccc
ggatttatggtatcatccgtgacggtctgaactgggttcgagataatttggcagagtaatcggatagattggtgataaaattctcccagggtagag
tcctaatgatcacctaaaactggctcagtttttcaaactctggaa*aataa*attcttaagaattttaaattcacctgtct

Vicugna pacos (GenBank: ABRR03000981.1)

MDTQRASLSLGRWSLWLLLLGLAVPWASIEALSYREAVLRAVDRLNEQSSDPNLYRLLELDLPPKAYEDLDAPKPVSFTVKETVCPRRTQLPPEQCA
FKENGLVKQCVGTITLDQVKGQMNIACDELQSI**GFFKKARKKLKNAWRKVGPIVGPLLNVFG** (VpCATH-29)

cactgcccagcaatcccctgaggcaagagcggggcctgaggcaactccagctcacaagctggctggggacagggatggggcaggtggggaaggctcc
tgcttgagcaattgcatcaggcccaggctgggc**ataaa**ggagggtcctgtgggctaggacgaggcagactcggggaccatggatacccagaggccca
gcctctccctgggccgctggtcactgtggctactactactgggactagcagtgccttgggccagcattgaggccctgagctatagggaggcggtgct
tcgagctgtggatcgcctcaatgagcagtcctcagaccccaatctctaccgcctcctggagctggacctgccgcccaaggctgtgagtcggggaggg
gctggggagggaggcgtctcccgccaggcttggccacactgtccctccctatgctcacgctgtacctcctgtcaggaaggcacctctccctctaagt
gggatcccacctctcacagaaatcctcccagagctgggtcccctctccttgtgagagtcttctgcctggcatctctgctgtgggaacagagccctga
cagctcgctcaggcttcaggggctcctgggagctccagggatacgctggggtcactggctctgggatgtgacttccctgcttccactcccctgtgca
ccccagtgtctccccaccagggaagagctgtgctcagcctggggtttccagtgacaaggtctttccctgcaggtgtcccgtgacttccctcagcccc
tccaagggtaggtgcccctcaggagagcaggtctgagttcccctcctgctccctgtgtgcctgggatcccaggaccaggctgtgtcctcctcccctc
tgcacccagcccccagcccagggccaggcacatgggactggctctcaaagggccttgtcctgctggggcagggagacaggtcacacaaaggagcatg
agcctgaatcagtctccccactttgatccttcagcagtacgaggacctagatgccccgaaacctgtgagcttcacggtgaaggagaccgtgtgtccc
aggaggacacagctgccaccggagcagtgtgccttcaaggagaatggggtgaggctgggggcttagggcgctggtgggtg **[gap61bp]** agacaggt
cacccaaggaagcatgagcctgagccagtctccccactttgatccttgagcaggacgaggacccagatgccccgaagcctgtaagctttatgttgaa
agagaccatgtgtcccaggaggacacagcagccaccggagcagtgtgccttcaaggagaaaggggtgaggctgggggcgctggtggatgcctcccaa
ggacccaaacaggaggctccaggagaaggcttgcagcccctggggtgaggctgggtagttatggctcaggggttccactgtgacctggaatatcccc
ttccagctggtgaaacagtgtgtggggacaatcactctggaccaggtcaagggccaaatgaacatcgcctgtgatgaggtgagtgagtccttccggg
ctgcaagggctgctagtggaggtggtgtgtggaacagcctttggaccagttcccctctgtgccatccctgtagcggcaggagagccctcctcccctg
gctcccccctcacccgagccccagatctccaggctgggctctgcaatcccttagagcagtggttctctcagtgggctccccacccaggacttgtcaga
aaggcagattctgtttccactgagacctactgaatcagagtctggggtgggacccagcaacttgtatttcaaccagctgaaggggattctgactgtg
ctaacatttgagaggcactgactagagtaatgggctttcagcccctgctcccctctggtttgctgggatgggcttgcgacccagagtagccccttgt
catctctggacctcgatttcctcatctgtgtgtgggtgtagggactcaaccacatgctccaaaagtcacagccagagggtgaactggggacacaagg
ctcctggggttacccagttaggggaggtgcccaggtgtggagatgtctggtctcacctctggttccattccccatatgtaacccggttctttctact
tcacagctccagagtattgggtttttaagaaggcacgtaaaaagcttaaaaacgcctggcggaaggttggcccaatagttggcccgttactcaatg
tatttgggtaaatcgggagcccatggaagagccctaagggtctgccggcattgcctcaagctactggactctgaga*aataa*agtcttgtgaaagcaa
cttcctgcagacttcagtttcatttgtctcacctcctcccatttactgagaccaaatccttaactctggagctgtcctgcgtatgtgtgtgtgtgtg
ggcgtgtggtcactc

Figure S4 (part 2). Structure of *CATHL* genes and corresponding preprocathelicidins of *Vicugna pacos*. Predicted intron/exon boundaries are highlighted in red. Exons are highlighted in green. Signal peptide sequences are marked in yellow/black. Signal peptide sequence identified with SignalP 5.0 (www.cbs.dtu.dk/services/SignalP/). Peptide sequences of putative mature cathelicidins are marked with red font. The TATA-box signal is in bold and grey marked and the polyadenylation signal is in italics and grey marked.

Vicugna pacos (GenBank: JEMW01026878.1)

METQRDSLSLRRWSLWLLLLGLAVPWASTEALSYREAVLRAVDRLNEQSSDPNLYRLLELDLPPKADEDLDAPKPVSFTVKETVCPRRTQLPPEQCA
FKENGVVKQCLGTVNLYQLRDNYDITCNELQSV**GFFGRIHDFFRDGVNWVRDKVGKVIGYIGDKIRPG** (VpCATH-35)

tcccctgaggcaagagcggggcctgaggcaactccagctcacaccctggccagggacagggatggggcaggtggggaaggctcctgcatgagcaatt
gcatcaggcccaggctgagc**ataaa**ggagggtcctgtgggctgggactaggcagactcgggaaccatggagacccagagggacagcctctccctgcg
ccgctggtcattgtggctactgctgctgggactagcggtgccttgggccagcactgaggccctgagctatagggaggcggtgcttcgagctgtggat
cgcctcaatgagcagtcctcagaccccaatctctaccgcctcctggagctggacctgccgcccaaggctgtgagtcggggaggggctggggagggag
gcgtctcccgccaggcttggccacactgtccctccctttgctcgcgctgtacctcctgtcaggaaggcacctctccctctaagtgggatcccacctc
tcacagaaatcctcccagagctgggtcccctcccgttgtgagagccttctgcctggcatctctgctgtgggaacagagccctgacagcccgctcagg
ctccagggcctcctggggggctccagggatacgctggggtcactggctctgggatgtgacttccctgcttccagtcccctctgcaccccattgtctcc
ccaccagagaagggttgtgctcagcctggagtttccagtgacaaggtctttccctgcaggtgtcccctgacttccctcagcccctccaaggggagct
gtccctcaggagtcagggtctgaggtcccctcctgctccctgtgcacccggaagccctggatcaggcacatgggacgggctgtcaaggtttcttgtc
ctgctggggcagggagacaggtaacacaaagaagcatgagcctgagtcagtctccccactttgatccttcagcaggacgaagacctagatgccccaa
agcctgtgagcttcacggtgaaggagaccgtgtgtcccaggaggacacagctgccaccggagcagtgtgccttcaaggagaatggggtgaggctggg
gattagggcgctggtgggtgcctcccaagacctgaacaggaggcttggggcgggtggtggttagcagctcctggggtgaggtgtggtgtttatggct
caggggttccagtgtgacctggagtctctccttccaggtggtgaaacagtgtttggggacagtcaacctttaccagctcagggacaactacgatata
acatgtaatgaggtgagtgaccccttctgggctgcaggggctgctggtggaggtggtgtgtggaacagcctttgggccaattcccctctgtgccatc
cctgtagcggcaggaaagccctcctcccctgactcccccctcacccaagccccaggtctccagccgggttctccaatcccttagagcagtggttctct
cagtgggctccccacttgggaactagagaaaaaaagctgattttcaggcctgaccagagctacggaatcactgtgtgagttggagcccagtaatttc
tattttatgagcccttctggggggattctgactgtgctgaagcgtgagaggcagtgactagagacaggacctttctgcccccgctcccgtccagcttt
tctgagatgggtttgtgcccctggaagccccctgtcatctctggaccttggtcctcatctgtctgtgggagtgatgattcaaccacgtgctccaagt
gacaaagccaaggaggtcaaatggtgcctcaagtctccttcggtggtccaagatggaagattttcaggtggggagggatctggtcttgtccctaagt
caatcctcacaagaatcctgtttctttctgctgcacagctccaaagtgtcgggttctttggccggattcatgatttcttccgtgatggtgtgaactg
ggttcgagataaggttggcaaagtaatcggatacattggtgataaaattcgcccagggtagagtcctaa *[contig end]*

Vicugna pacos (GenBank: ABRR03000981.1)

METQRASLSLRRWSLWILLLGLAVPWASAQALSYREAVLRAVDRLNEQSSDPNLYRLLELDLPPKADEDLDAPKPVSFTVKETVCPRRTQLPPEQCD
FKEKGVVKLCLGTVTLDQVKDQIDISCNKLQSV**RRFFPRPFPPGPFPFPPFPPIPPIPPFPFPGPIIR**GRR (VpCATH-38)

agatggagaaaccatttcttcaccttgcacaggaggcagacctcacccactgccagcaatcccctgaggcaagagcggggcctgaggcaagtccagc
tcacaccctggccgggggcagggatggggcaggtggggaaggctcctgcttgagcaattgcatcaggcccaggctgagc**ataaa**ggagggtcctgtg
ggctgggaggtggcagactcggggaccatggagacccagagggccagcctctccctgcgccgctggtcactgtggatactgctgctgggactagcgg
tgccttgggccagcgcccaggccctgagctatagggaggcggtgcttcgagctgtggatcgcctcaatgagcagtcctcagaccccaatctctaccg
cctcctggagctggacctgccgcccaaggctgtgagtcggggaggggctggggagggggggcgtctcccgccaggcttggccacactgtccctccctt
tgctcgcgctgtacctcctgtcaggaaggcacctctccctctaagtgggatcccacctctcacagaaatcctcccagagctgggtcccctcccgttg
tgagagccttctgcctggcatctctgctgtgggaacagagccctgacagcccgctcaggctccagggactcctgggagctccagggatacgctgggc
tcactggctctgggatgtgacttccctgcttccagtcccctgtgcaccccagtgtctccccaccagggaagggctgtgctcagcctaggggttccag
tgactaggtctttccctgcaggtgtcccctgacttccctcagcccctccaagagggaggtgcccctcaggagaggggtctgaggtcccctccttct
ccctgtgtacctgggagcccaggtccaggctgtgtcctcttcccctctgctcccagcccccagcccagggccaggcacatgggacggtctttcaaga
gggctgttctgctgtggggaagggagacaggtcacccaaggaagcatgagcctgagccagtctccccactttgatccttcagcaggacgaggaccta
gatgccccaaagcctgtgagcttcacggtgaaggagaccgtgtgtcccaggaggacacagctgccaccggagcagtgtgacttcaaggagaaaggggg
tgaggctgggagctgtgaacgctggcggatgcgtcccaaggacccgaacaagaggctccggagggtggttagcagcccctggggtgaggctgtgtgt
ttatggctcaggattccagtctgaactagaggtttcccttccaggtggtgaaactgtgtttggggacagtcactctggaccaggtcaaggaccaaa
tagacatcagttgtaataaggtgagtggcccttcttgttttgagctgctccggggagaggcgtggaacatcatttggaccagttttgttctttttca
ttcagggcttcccaccctggctcccccatcacctgagcccaagtccccagcctgggctctgcaatcccttagagcagtggtttgttctctaagtggc
attctcacctgggaactagatggaaaggcaggttcttagtccacattcagacctactgagacaaagttggggtgggtcccagaaatttgtattttaa
gaagccctcctggggattctgactgtgctgaagtttgagaggcactgactacagtaatgggatttctgcctctgcttctgtccagctgtggtggcca
gtgatccggggaggccttggcatctctgtgtcctggttttctcatctgtctatgggagtagggattcaaccacatgctccaaacttatagccacagg
gtgaatgggacccaagcctccaggagtggctcaggagtaggatgtgtagaaaggacctgattcatttgattcaggatcagtactgacaagaagcc
tgtttcttcctggttcacagctccagagtgtaaggcgatttttccacgaccatttccacctggacccttcccatttccccctttcccaccaatccc
ccctataccaccatttccatttcctggccgattattagaggacgacggtgaaggattggctgataccttgcccatcagaggattctgttgaattgt
gagcccagggaagtcctaagggtcttctttattctggctcagtgtttcagattctga*aataa*attgttctgaaaacaacttcctccaggtatctccc
acttactgtgaccaggtcctaaactctgggagccgagtgtgtgtgtgggtgtgtgcaagcgtgtgtttgcgtgtgtgtgtatcagtgaatgtggtca
tttccgtgcgca

Figure S4 (part 3). Structure of *CATHL* genes and corresponding preprocathelicidins of *Vicugna pacos*. Predicted intron/exon boundaries are highlighted in red. Exons are highlighted in green. Signal peptide sequences are marked in yellow/black. Signal peptide sequence identified with SignalP 5.0 (www.cbs.dtu.dk/services/SignalP/). Peptide sequences of putative mature cathelicidins are marked with red font. The TATA-box signal is in bold and grey marked and the polyadenylation signal is in italics and grey marked.

Vicugna pacos (GenBank: JEMW01005218.1)

METQRDSLSLRRWSLWLLLLGLAVPWASAQALSYREAVLRAVDRLNEQSSDPNLYRLLELDLPPKADEDPDAPKPVSFTVKETVCPRKTQQPPEQCA
FKENGVVKQCVGTVTLDQSRDQQDITCDELQSV**RRIRRPRLPRPRVPRPRIPPRIPRPVLPPPRVPFPRFPR**GR (VicBac)

gagcctgaggcaagtccagctcacaccctggccaggggcagggatggggcaggtggggaaggctcctgcttgagcaattgcaccaggcccaggctgg
gc**ataaa**ggagggtcctgtgggctgggaggaggcagactcggggaccatggagacccagagggacagcctctccctgcgccgctggtcactgtggct
actgctgctgggattagcggtgccttgggccagcgcccaggccctgagctatagggaggcggtgcttcgagctgtggatcgcctcaatgagcagtcc
tcagaccccaatctctaccgcctcctggagctggacctgccgcccaaggctgtgagtcggggaggggctggggagggaggcgtctcccgccaggctt
ggccacactgtccctccctttgctcgcgctgtacctcctgtcaggaaggcacctctccctctaagtgggatcccacctctcacagaaatcctcccag
agctgggtcccctcctgttgtgagagccttctgcctggcatctctgctgtgggaacagagccctgacagcccgctcaggctccagggcctcctgggg
gctccagggatacgctggggtcactggctctgggatgtgacttccctgcttccagtcccctgtgcaccccattgtttccccaccagggaagggctgt
gctcagcctggggttccagtgacaaggtcttcccctgcaggtgtcccctgacttccttcagcccctccaaggggagctgtccctcaggagagtggg
tctgagttcccctcctgctccctgtgtgccttggagcccaggaccaggctgtgtcctcctcccctctgcacccagcccgcagcccaggatcaggaac
atgggaactgctttcaagggtgattgtcctgctggggcagggagacaggtcacacaatgatgcatgagcctgagccagtctccccactttgatcctt
gagcaggacgaggacccagacgccccgaagcctgtgagcttcacagttaaggagaccgtgtgtcccaggaagacacagcagccaccggagcagtgtg
ccttcaaggaaaatggggtgaggctgggggcttagggcgctggtaggtgcctccaaagaacaggaacaggaggctcgaagaggggggtgttatttgca
gcccctggggtgaggctgggtggttatggctcaggggttccagtgtgacctgatgtctccccttccaggtggtgaaacagtgtgtggggacagtcac
cttggaccagtccaggaccaacaggatatcacctgtgatgaggtgagtgaccccttccaggctgcagaggctgctgctgggtctggtgtgtggaac
atcctttggaccatttcccctctgtgccatccctgtagcggcaggaaagccatcctcccctggctccccctcacctgaaccccaggtctccaggccg
ggctctgcaatcccttagagcagtggttctctcagtaggctccccacacaggacgtgtcagaacggcaaaaattctcagtccccactctgacgtact
gaatcagagtctaggttggggcccagtaatttgtcttataagagcgcttctaagggattctgtgctgaagtttaagaggcactgactagagtagcgg
ctttcagcccctgcttctgtccttcattgctgggaagggcttgtgcccctgggaagccccttgtcatctctgggccttggcttcctcctctgtgagt
gagtgtagggattcaaccatgtgctccaaaggtcacagccagagggtgaactggggcctcagggcacctggggtggcactgggaggggggatcaccag
gtgaggacacaggggtcttgggttgaccctggttccgacccagaagaaatctgttttcctcctggttcacagcttcagagtgttaggagaattcgcc
ggccacggctgccaaggccaagggtgccacgtccacgcatcccaccaaggataccaaggccagtattgccgccaccaagggttcctttccaaggtt
tccgagaggacggtgaatggttggctgataactctcccataagggccgaaggaagagtacagagggtctgcctgccccgcgtcaggacctggacacc
aaaag*ataaa*ttttatgaaaaaaactttctccaagcttctattttaattgtgtcacctgatcacacacacagggaccaaatccac

Vicugna pacos (GenBank: ABRR03000981.1)

METQRASLSLRRWSLWLLLLGLAVPWASAQALSYREAVLRAVDRLNEQSSDPNLYRLLELDLPPKADQDPDAPKPVSFTVKETVCPRRTQQPPEQCA
FKKNGLVKQCVGTVTLDQSKDQQDITCDKARRSRPLPFLSPRIPRQ**SFPPPNFPPPNFPPPEFPPPNFPPPNFPPPRFPPPNFPPPNFPPPEFPLQN**
FPPPI (Vp-prophenin)

gcaatcccctgaggcaagagcggggcctgaggcaagtccagctcacaccctggccgggggcagggatggggcaggtggggaaggctcctgcttgagc
aattgcatcaggcccaggctgagc**ataaa**ggagggtcctgtgggctgggacgaggcagactcggggaccatggaaacccagagggccagcctctccc
tgcgccgctggtcactgtggctactgctgctgggactagcggtgccttgggccagtgcccaggccctgagctatagggaggcggtgcttcgagctgt
ggatcgcctcaatgagcagtcctcagaccccaatctctaccgcctcctggagctggacctgccgcccaaggctgtgagtcggggaggggctggggag
ggaggcgtctcccgccaggcttggccacactgtccctccctttgctcgcgctgtacctcctgtcaggaaggcacctctccctctaagtgggatccca
cctctcacagaaatcctcctagagctgggtcccctcccgttctgagagccttctgcctggcatctctgctgtgggaacagagccctgacagctcgct
caggctccagggcctcctggggctccagggatacagttgggtcactggctcttgggtgtgacttccctgcttccagtcccctgtgcaccccattgt
ctcccaattagggaagggctgtgctcagcctggatgttccagtgacaaggtcttcccctgcaggtgtcccctgacttccctcactcgtctaagggg
agctgcccctcaggagagcgggtctgaggtcccctcctgctccctgtgtcccttggagcccaggaccaggctgtgtcctcctcccttctgcacccag
cccgcagtccagggccaggcacatgggaggggttgtcaaggggctgtcctgctgggggcagggagataggtcatcaaggaagtatgagcctgagcc
agtctccccactttgatccttgagcaggaccaggacccagatgccccaaagcctgtgagcttcacggtgaaggagaccgtgtgtcccaggaggacac
agcagccacctgagcagtgtgccttcaagaagaatggggtgaggctgggggcactggcggatgcctcccaaggacctgaataggagtgtccaaagaa
gccttgcagcccctggggtgaggctgggtggttgtggttcagggcttccagtctgacctggagtctccccttctagctggtgaaacagtgtgtgggg
acagtcactctggaccagtccaaggaccaacaggacatcacctgtgataaggttaatgactccttctgggctgtaggggctgcttgtaggagtgggg
tgtggaacatcttttggaccaattcccttctgtgccatcactttaagggggaaattcctcctcccctggctccccctcaccagagccccaggtctc
caggctgggctctggtatcccttatagcagtggttctctcagtgtcctccccacttggaaactagagaaaacggctgattctctggccccagtgaga
tctacccaattagttggaggtgggtgccagcaattcttattttaaggcttcctgggcatcctgactacttgaacgacactgactagagtaatggact
ttctgcccctgctcccatccagctttgctgggatgggcttgtgcccctgggaaggcccttatcatctctggaaccagtgtcctcatctgtgcgtggg
tgaggtgatatgctcctaatgtcacaggcagaggtgaactggggacccaagtttcctggagtccacctggaaagtggttcagtgtagagagaagggc
cttgtgattgtggcccagttagtacaagaaaactttcttttgatccacaggcccggagatctcgcccgctaccctttctatcgccgaggataccacg
gcagagtttccctccgccaaatttccctccgccaaatttccctcctcctgagtttcctccaccaaatttccctccgccaaatttccctcctcctcgg
tttcctccgccaaatttccctccgccaaatttccctcctcctgagtttcctctgcaaaatttccccccacccatctaattaccccctgccaaatttgc
attcccatccagccctccaagatttgctacaagaaggtgaaagatttacagataactcgtgtataagggcatttagcgaatcagaagcccggggaga
ctcctaagggtctgctttgccctgggtcaggatttgaag*aataa*attcctgtgaaaacactttgctccaggcttcagttttacttttctc

Figure S4 (part 4). Structure of *CATHL* genes and corresponding preprocathelicidins of *Vicugna pacos*. Predicted intron/exon boundaries are highlighted in red. Exons are highlighted in green. Signal peptide sequences are marked in yellow/black. Signal peptide sequence identified with SignalP 5.0 (www.cbs.dtu.dk/services/SignalP/). Peptide sequences of putative mature cathelicidins are marked with red font. The TATA-box signal is in bold and grey marked and the polyadenylation signal is in italics and grey marked.

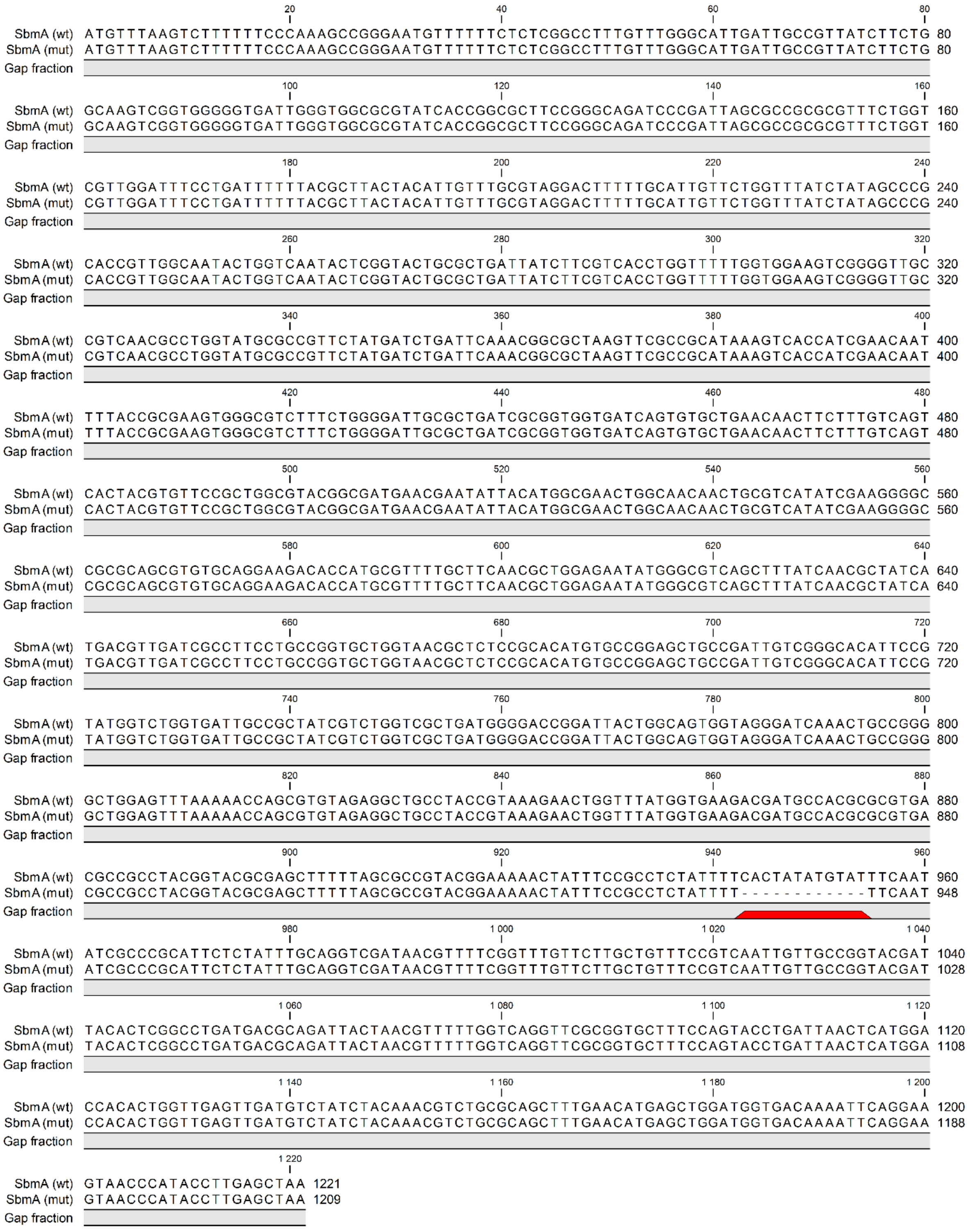

Figure S5. Alignment of nucleotide sequences of genes encoding SbmA from *E. coli* MDR CI 1057 "wt" (wild type) and mutant SbmA from VicBac[1-22]-resistant *E. coli* MDR CI 1057 "mut" obtained after selection. Nucleotide deletion is highlighted with red.

Table S1. Amino acid sequences and molecular masses of the peptides used in this study

Peptide	Origin	Sequence	Molecular mass, Da	
			Calculated [M+H]$^+$ value[1]	Measured value[2]
Bac7[1-22]	Rec	RRIRPRPPRLPRPRPRPLPFPR	2783.72	2783.89
PR-39[1-22]	Rec	RRRPRPPYLPRPRPPPFFPPRL	2765.61	2765.35
VicBac[1-14]	Rec	RRIRRPRLPRPRVP	1825.17	1824.96
VicBac[1-16]	Rec	RRIRRPRLPRPRVPRP	2078.32	2078.05
VicBac[1-18]	Rec	RRIRRPRLPRPRVPRPRI	2347.51	2347.25
VicBac[1-20]	Rec	RRIRRPRLPRPRVPRPRIPP	2541.61	2541.52
VicBac[1-22]	Rec	RRIRRPRLPRPRVPRPRIPPRI	2810.80	2810.84
VicBac[1-39]	Synt	RRIRRPRLPRPRVPRPRIPPRIPRPVLPPPRVPFPRFPR*	4815.98	4815.70
Melittin	Synt	GIGAVLKVLTTGLPALISWIKRKRQQ	2846.74	2846.60
LL-37	Synt	LLGDFFRKSKEKIGKEFKRIVQRIKDFLRNLVPRTES	4491.58	4491.40

Rec – recombinant peptide

Synt – synthetic peptide

* *C*-terminal amidation

[1] according to Expasy Isotopident tool

[2] monoisotopic m/z were measured using MALDI-TOF MS

Table S2. Amino acid sequences of natural cathelicidins from *Camelidae* species

Animal	Sequence	GenBank ID
**Vp22-like cathelicidins**		
Camelus dromedarius	KIINLPWRPPPRKRPIRVIYV	JWIN01013794.1<br>LSZX01138262.1
Camelus ferus	KIINLPWRPPRRKRPIRVIYV	AGVR01057826.1
Camelus bactrianus	KIINLPWRPPRRKRPIRVIYV	JARL01035148.1
Vicugna pacos	KVINLLWRPPPRRQRPPRLIYV	JEMW01023459.1
Vicugna vicugna	KVINLLWRPPPRRQRPPRLIYV	PNXW01017670.1<br>JEMW01026878.1
Lama guanicoe	KVINLLWRPPPRRQRPPRLIYV	PNXV01010685.1
Lama glama	KVINLLWRPPPRRQRPPRLIYV	PNXU01026596.1
**23-residue cathelicidins (MAP-like)**		
Camelus dromedarius	GLFGPILGIVRAGVKLVLDKFGK	JWIN01033265.1<br>JDVD01079641.1
Camelus bactrianus	GLFGPILGIVRAGVKLVLDKFGK	JARL01035147.1
Lama guanicoe	GILGPILRIVAAGLDWLQHKRGK	PNXV01004884.1
Lama glama	GILGPILRIVAAGLDWLQHKRGK	PNXU01036743.1
**Vp24-like cathelicidins (MAP-like)**		
Camelus dromedarius	GLGRIVRKAIKYVPTVIRIASWFL	LSZX01137296.1<br>JWIN01015089.1
Camelus ferus	GLGRIVRKAIKYVPTVIRIASWFL	AGVR01002349.1
Camelus bactrianus	GLGRIVRKAIKYVPTVIRIASWFL	JARL01005658.1
Vicugna pacos	GFGRIVRKAIKYVPTVIRIASWFL	ABRR02174663.1<br>JEMW01020494.1<br>ABRR02172633.1
Vicugna vicugna	GFGRIVRKAIKYVPTVIRIASWFL	PNXW01014781.1
Lama guanicoe	GFGRIVRKAIKYVPTVIRIASWFL	PNXV01005610.1
Lama glama	GFGRIVRKAIKYVPTVIRIASWFL	PNXU01002030.1
**Vp26-like cathelicidins (MAP-like)**		
Vicugna pacos	RSFPRIYGIIRDGLNWVRDNFGRVIG	JEMW01026878.1
Vicugna vicugna	RSFPRIYGIIRDGLNWVRDNFGRVIG	PNXW01021834.1
Lama guanicoe	RSFPRIFGIIRDGLNWVRDKLGRVIG	PNXV01022927.1
Lama glama	RSFPRIFGIIHDGLNWVRDKLGRVIG	PNXU01023268.1
**Vp29-like cathelicidins (MAP-like)**		
Camelus dromedarius	GFFKKARNKLKNAWRKVGPIVGPLLTFFG	JWIN01010394.1<br>LSZX01137298.1
Camelus ferus	GFFKKARNKLKNAWRKVGPIVGPLLTFFG	AGVR01002349.1
Camelus bactrianus	GFFKKARNKLKNAWRKVGPIVGPLLTFFG	JARL01005658.1
Vicugna pacos	GFFKKARKKLKNAWRKVGPIVGPLLNVFG	JEMW01020494.1<br>ABRR02172634.1
Vicugna vicugna	GFFKKARKKLKNAWRKVGPIVGPLLNVFG	PNXW01014781.1
Lama guanicoe	GFFKKARKKLKNAWRKVGPIVGPLLNVFG	PNXV01005610.1
Lama glama	GFFKKARKKLKNAWRKVGPIVGPLLNVFG	PNXU01002030.1
**Vp38-like cathelicidins**		
Camelus dromedarius	RGFFPPPFPPGPFPFPPFPPIPPIPPFPFPRPFVRRRR	JDVD01002168.1<br>JWIN01034589.1
Camelus ferus	RGFFPPPFPPGPFPFPPFPPIPPIPPFPFPRPFVRRRR	AGVR01053365.1
Camelus bactrianus	RGFFPPPFPPGPFPFPPFPPPPPIPPFPFPRPFVRRRR	JARL01035147.1
Vicugna pacos	RRFFPRPFPPGPFPFPPFPPIPPIPPFPFPGPIIRGRR	JEMW01019668.1

		ABRR02179311.1
Vicugna vicugna	RRFFPRPFPPGPFPFPPFPPIPPIPPFPFPGPIIRGRR	PNXW01021834.1
Lama guanicoe	RRFFPGPFPPGPFPFPPFPPIPPIPPFPFPGPIIRGRR	PNXV01010688.1
Lama glama	RRFFPGPFPPGPFPFPPFPPIPPIPPFPFPGPIIRGRR	PNXU01023268.1
**Bac7-like cathelicidins (VicBac, CamBac, LamBac)**		
Camelus dromedarius	RSIRRPRLPRPRVPRPYIPPRIPRPVLPPPRFPIPRFPRGR	JDVD01078062.1
Camelus ferus	RSIRRPRLPRPRVPRPYIPPRIPRPVLPPPRFPIPRFPRGR	AGVR01053366.1
Camelus bactrianus	RSIRRPRLPRPRVPRPYIPPRIPRPVLPPPRFPIPRFPRGR	JARL01035149.1
Vicugna pacos	RRIRRPRLPRPRVPRPRIPPRIPRPVLPPPRVPFPRFPRGR	JEMW01005218.1<br>ABRR02002833.1
Vicugna vicugna	RRIRRPRLPRPRVPRPRIPPRIPRPVLPPPRVPFPRFPRGR	PNXW01021835.1
Lama guanicoe	RRIRRPRLPRPRVPRPHIPPRIPRPVLPPPRVPFPRFLRRR	PNXV01010685.1
Lama glama	RRIRRPRLPRPRVPRPHIPPRIPRPVLPPPRVPFPRFLRRR	PNXU01024686.1
**Prophenin-like cathelicidins**		
Camelus dromedarius	RFPVPNFPPPNFPPPQFPPPNFPPPRFPPPNFPPPNFPPPE<br>FPLPNFPPPNFPPPEFPLPNFPPPI	JDVD01076911.1
Camelus dromedarius	RFPAPNFPPPNFPPPEFPLPNFPPPNFPPPEFPLPNFPLPN<br>FPPPNFPPPQFPPPNFPPPRFPPPNFPPPNFPPPEFPLPNF<br>PPPNFPPPEFPLPNFPPPI	JWIN01034417.1<br>LSZX01137301.1
Camelus ferus	RFPAPNFPPPNFPPPRFPPPNFPPPNFPPPNFPPPEFPLPN<br>FPPPNFPPPEFPLPNFPPPI	AGVR01059010.1
Camelus bactrianus	RFPAPNFPPPNFPPPRFPPPNFPPPNFPPPNFPPPEFPLPN<br>FPLPNFPPPNFPPPRFPPPNFPPPNFPPPNFPPPEFPLPNF<br>PPPNFPPPEFPLPNFPPPI	JARL01005656.1
Vicugna pacos	SFPPPNFPPPNFPPPEFPPPNFPPPNFPPPRFPPPNFPPPNF<br>PPPEFPLQNFPPPI	ABRR02179514.1
Vicugna vicugna	SFPPPNFPPPNFPPPEFPLQNFPPPI	PNXW01021834.1
Vicugna vicugna	SFPPPNFRPPRFPPPNFPPPRFPPPNFPPPRFPPPNFPPPRF<br>PPPDFPPPRFPPPDFPPPRFPPPNFPPPRFPPPNFPPPNFPP<br>PEFPPPNFPPPNFPPPNFPPPEFPLQNFPPPI	PNXW01017671.1
Lama guanicoe	SFPPPNFRPPRFPPPNFRPPRFPPPNFPPPRFPPPNFPPPRF<br>PPPNFPPPNFRPPNFPPPRFPPPNFPPPEFPPPNFPPPNFR<br>PPNFPPPEFPLQNFPPPI	PNXV01010685.1
**Vp35-like cathelicidins (MAP-like)**		
Camelus dromedarius	GLFGRIRDFFHDGVNWVRDKVGKVIGYIGDKIRPG	JWIN01007525.1
Camelus dromedarius	GLFGRIRDSIRNRVNRVRDAIGKVIGYVGDKIRPG	
Camelus ferus	GLFGRIRDFFHDGVNWVRDKVGKVIGYIGDKIRPG	AGVR01065784.1
Camelus bactrianus	GLFGRIRDFFHDGVNWVRDKVGKVIGYIGDKIRPG	JARL01035150.1
Camelus bactrianus	GLFGRIRDSIRKRVNRVRDAIGKVIGYVGDKIRPG	JARL01005655.1
Vicugna pacos	GFFGRIHDFFRDGVNWVRDKVGKVIGYIGDKIRPG	JEMW01026878.1<br>ABRR02002839.1
Vicugna vicugna	GFFGRIRDFFRDGVNWVRDKVGKVIGYIGDKIRPG	PNXW01021834.1
	GVFGRIRNFFRDRVNRVRDTVGKVIGYIGDKIRPG	PNXW01021834.1
Lama guanicoe	GFFGGIRDFFRDGVNRVRDTVGKVIGYIGDKIRPG	PNXV01022928.1
	RLFGGIRDFIRDRVNRVRDKVGKVIGYIGDKIRPG	PNXV01010688.1
Lama glama	GFFGGIRDFFRDGVNRVRDTVGKVIGYIGDKIRPG	PNXU01023268.1
